# Supplementary material for: Web-based support for self-management strategies versus usual care for people with COPD in primary healthcare: a protocol for a randomised, 12-month, parallel-group pragmatic trial
Source: BMJ Open. 2019 Oct 7;9(10):e030788. doi: 10.1136/bmjopen-2019-030788 (PMC6797375; doi:10.1136/bmjopen-2019-030788)
Supplement: Author's manuscript [file bmjopen-2019-030788.draft_revisions.pdf]

BMJ Open

BMJ Open is committed to open peer review. As part of this commitment we make the peer review history of every article we publish publicly available.

When an article is published we post the peer reviewers' comments and the authors' responses online. We also post the versions of the paper that were used during peer review. These are the versions that the peer review comments apply to.

The versions of the paper that follow are the versions that were submitted during the peer review process. They are not the versions of record or the final published versions. They should not be cited or distributed as the published version of this manuscript.

BMJ Open is an open access journal and the full, final, typeset and author-corrected version of record of the manuscript is available on our site with no access controls, subscription charges or pay-per-view fees (http://bmjopen.bmj.com).

If you have any questions on BMJ Open's open peer review process please email info.bmjopen@bmj.com

BMJ Open

Web-based support for self-management strategies versus usual care for people with COPD in primary healthcare: a protocol for a randomised, 12 months, parallel-group pragmatic trial.

Journal:	*BMJ Open*
Manuscript ID	bmjopen-2019-030788
Article Type:	Protocol
Date Submitted by the Author:	01-Apr-2019

Complete List of Authors:	Stenlund, Tobias; Umea University Department of Community Medicine and Rehabilitation, Physiotherapy<br>Nyberg, André; Umea University Department of Community Medicine and Rehabilitation, Physiotherapy<br>Lundell, Sara; Umea University Department of Community Medicine and Rehabilitation, Physiotherapy<br>Wadell, Karin; Umea University Department of Community Medicine and Rehabilitation, Physiotherapy

Keywords:	Pulmonary Disease, Chronic obstructive, eHealth, self-management strategies, PRIMARY CARE

SCHOLARONE™
Manuscripts

Web-based support for self-management strategies versus usual care for people with COPD in primary healthcare: a protocol for a randomised, 12 months, parallel-group pragmatic trial.

AUTHORS

Tobias Stenlund, PhD, Dept. of Community Medicine and Rehabilitation, Physiotherapy, Umeå University, Umeå, 90187 Sweden. Tel.: +46907868040. E-mail: tobias.stenlund@umu.se

André Nyberg, Associate Professor, Dept. of Community Medicine and Rehabilitation, Physiotherapy, Umeå University, Umeå, 90187 Sweden. E-mail: andre.nyberg@umu.se

Sara Lundell, PhD, Dept. of Community Medicine and Rehabilitation, Physiotherapy, Umeå University, Umeå, 90187 Sweden. E-mail: sara.lundell@umu.se

Karin Wadell, Professor, Dept. of Community Medicine and Rehabilitation, Physiotherapy, Umeå University, Umeå, 90187 Sweden. E-mail: karin.wadell@umu.se

Corresponding author: PhD Tobias Stenlund Dept. of Community Medicine and Rehabilitation, Physiotherapy, Umeå University, Umeå, 90187 Sweden. Tel.: +46907868040. E-mail: tobias.stenlund@umu.se

Keywords: Pulmonary Disease, Chronic obstructive; eHealth; primary care; self-management strategies

Word Count: 3900

For peer review only

ABSTRACT

Introduction

The use of adequate self-management strategies for people with chronic obstructive pulmonary disease (COPD) may increase the level of physical activity (PA), improve health-related quality of life (HRQoL) and reduce healthcare use. Whether a web-based solution in addition to prompts (e-mail and SMS) could be used to promote self-management strategies to facilitate behavior change in people with COPD are contradictory, and so far only a pilot study has been performed in Sweden. This clinical trial aims to generate evidence on the effect of a web-based site, the COPD Web, in a cohort of people with COPD in a primary healthcare context.

Methods and analysis

The overall design is a pragmatic randomised controlled trial with pre- and post-assessments (3 and 12 months) and with a user experience evaluation. People with a diagnosis of COPD, treated in primary healthcare will be eligible for the study. A total of 144 participants will be enrolled by healthcare professionals at included primary healthcare centers and, after fulfilled baseline registration, randomised to either control or intervention group. All participants will receive usual care, a pedometer and a leaflet about the importance of PA. Participants in the intervention group will, also, get access to the COPD Web, an interactive self-managed web site that aims to support people with COPD in self-management skills. Participants in the intervention group will also continuously be supported by prompts that aim to encourage behavior changes.

The effect of participants' PA, dyspnea, COPD related symptoms, HRQoL, and health economics about healthcare use will be assessed using accelerometer and questionnaires. To identify enablers and barriers for the use of a web-based solution like the COPD Web to change behavior, semistructured interviews will be conducted in a subgroup of participants at the three months follow up.

Ethics and dissemination

Ethical approval has been received from the Regional Ethical Review Board in Umeå, Sweden. Dnr 2018-274-31. Findings will be presented at conferences, submitted for publication in peer-reviewed journals and presented to the involved healthcare professionals, participants and patient organisations.

Trial registration number

ClinicalTrials.gov: NCT03746873

For peer review only

Article Summary

Strengths and limitations of this study

- The use of the COPD Web will be automatically collected and analysed throughout the full intervention period, which will increase the understanding of the link between use of the COPD Web and the possible effects.
- Physical activity level will be objectively measured and bring knowledge about both short-term and long-term effects of using the COPD Web.
- The pragmatic design with generous inclusion criteria and many recruiting primary healthcare centres could enhance recruitment rates.
- Prompts will be sent continuously as a reminder and strategy to encourage greater exposures to the COPD Web.
- One limitation is that the sample size is large enough for analysing the effect on physical activity level but may not be large enough for all secondary outcomes.

For peer review only

Introduction

Background and rationale

Chronic obstructive pulmonary disease (COPD) is a chronic and disabling disease with substantial morbidity and mortality. The disease has a steady increase in prevalence and is now the third leading cause of death worldwide.[1] The high prevalence places a considerable burden on the healthcare system with a total yearly cost of COPD in Sweden estimated to 13.9 billion SEK[2] and the mean annual total costs for each person with COPD is 67% higher compared to a person without COPD.[3]

The symptom burden of the disease; respiratory symptoms as progressive dyspnea, fatigue, impaired physical performance, decreased level of physical activity (PA) and health-related quality of life (HRQoL) in people with COPD[4] is not only a consequence of the underlying condition, but depend also on the individuals' adaptation to the illness and their ability to manage their disease.[5 6]

Self-management strategies, including strategies to promote self-efficacy by increasing the individual's knowledge and skills and their confidence in successfully managing their disease, is therefore now an essential part of COPD management.[5]

This have shown to reduce breathlessness and impact of COPD in daily life, increase physical performance, level of PA, HRQoL, adherence to medication, as well as improve time to recovery after acute exacerbations and reduce overall health-related costs.[5 7 8] An increased level of PA is of utmost importance since PA has been shown to be decreased in all stages of the disease and degree of PA is considered the strongest predictor of all-cause mortality in people with COPD.[9 10]

Despite that treatment guidelines and literature strongly supports that non-pharmacological treatment (i.e., education, self-management strategies, exercise training)[11] should be provided, a vast majority of people with COPD are still excluded from these activities.[12 13] Web-based solutions are promising means of delivering health service, and may increase level of PA[14] as well as reduced use of health services.[15] However, studies evaluating whether a web-based solution as the COPD Web could be used to promote self-management strategies to support increased PA in people with COPD are contradictory.[16-18] The COPD Web is a web-based site, developed by our research group in co-creation with people with COPD, their relatives, healthcare professionals in primary healthcare (PHC) and researchers.[19] In a pilot study on 83 people with COPD[20 21] promising results with the increased self-reported level of PA were shown. To know whether this is true also for a larger COPD population an adequately powered randomised controlled trial is needed.

For peer review only

Objectives

The main aim is to generate evidence on the effect of the COPD Web in a cohort of people with COPD, currently enrolled for usual care within the PHC context in Sweden. This is of importance, as the vast majority of people with COPD are treated within PHC.[11 13] The specific aims are to evaluate the effect of the use of the COPD Web in an adequately powered group of people with COPD in PHC context, regarding i) level of PA; ii) dyspnea iii) HRQoL, iv) COPD related symptoms, v) health economics in relation to healthcare use; and vi) to identify enablers and barriers for the use of an eHealth solution like the COPD Web in order to change behavior.

We hypothesise that access and use of COPD Web, in comparison to usual care, will:

i) increase level of objectively measured PA in people with COPD,
ii) decrease dyspnea,
iii) increase disease-specific HRQoL,
iv) decrease number of and/or severity of COPD-related symptoms, and
v) decrease number of COPD-related healthcare contacts in PHC.

Methods and analysis

Trial design

The design is a pragmatic randomised controlled trial with pre- and post-assessments (3 months and 12 months) and with a user experience evaluation. The user experience evaluation is a necessary complement that will be performed to understand more about enablers and barriers for behavior change using web-based solutions like the COPD Web. The study is designed as a pragmatic trial[22] meaning that healthcare professionals, primarily COPD nurses, are involved in recruiting participants, the access to the intervention (COPD Web) is given by the researchers, but the intervention itself only uses self-instructional material and prompts (SMS and email). This design aims to minimise the effort from healthcare professionals and increase the possibility of self-management for people with COPD to improve the applicability of the findings to other healthcare settings. The protocol complies with the SPIRIT (Standard Protocol Items: Recommendations for Interventional Trials) recommendations for protocol reporting[23 24] checklist (Additional file 1) and the study will be reported according to CONSORT (Consolidated Standards of Reporting Trials) guidelines for pragmatic trials[22] and eHealth.[25]

Patient and Public Involvement (PPI)

We did not directly include PPI in this study, but our research group in co-creation with PPI developed the COPD Web used in the study.

For peer review only

Participants and intervention

Study settings

Primary healthcare centers (PHCCs) from different County Councils in Sweden, will constitute the study sites. The number of PHCCs are not limited; consequently, more PHCCs may be included during the study. At present 23 PHCCs are included, 11 centers situated in urban areas and 12 centers located in smaller cities or rural areas. The number of enrolled citizens at the included PHCCs range between 5,700 and 20,300 citizens. One of the included PHCC has no enrolled citizens but act as a rehabilitation unit that treats patients with a referral from other PHCCs. Following the majority of all healthcare services in Sweden, most of the included PHCCs are publicly funded, although private alternatives with an agreement with the County Council are also included.

Eligibility criteria

The trial will be conducted from 15 November 2018 until 144 participants are included. All people with a diagnosis of COPD (ICD-10:J44:9) who visit involved PHCCs due to their COPD will be eligible for inclusion in the study if they 1) can read and understand Swedish, 2) have a smartphone, tablet or computer with access to internet, 3) don't have dementia or other psychiatric condition that can prevent understanding of the intervention, 4) don't have severe comorbidity that can be considered as the contributing factor for limitation in PA, and 5) don't already use the COPD Web. In the case of exacerbation, the participant has to wait six weeks from the start of pharmacological treatment, before being eligible in the study.

Participant timeline

The recruitment begins at included PHCCs. To facilitate the recruitment of participants, the number of included PHCCs will not be restricted to nor the PHCCs size, location, how they are funded or the type of care and rehabilitation that the center offers. However, written consent from the operational manager at each PHCC has to be fulfilled before recruitment can start.

To increase the possibility of recruiting participants, the number of exclusion criteria are kept to a minimum. The recruitment will take place during the participant's regular visits at the PHCC where healthcare professionals will give information about the study. People with COPD interested in participation will have their contact information and results from latest pulmonary function test (not older than six months otherwise a new function test will be taken) sent to the research group as displayed in table 1 (t^{-1}). A researcher (TS) will after verbal agreement send questionnaires, informed consent form and activity monitor for baseline registration to the participants' homes (t^{0}). When the written informed consent and the baseline registration is fulfilled, the participants' are included and randomised to either the control or intervention group (t^{1}). Follow-up measurements with

questionnaires and activity monitor will be conducted at three months (t^2) and 12 months (t^4) after inclusion. A semi-structured interview will be done after the three months follow up (t^3) among a convenient sample in the intervention group.

The participants will be contacted by phone before every assessment (t^0, t^2, t^4) to ensure a suitable date for the activity monitoring. In case of non-response after any evaluation (t^0, t^2, t^4) the participant will be reminded by phone or/and email after two weeks and again after four weeks. These precautions will be made to maintain the participant in the study and increase the number of complete follow-ups.

Intervention

The COPD Web consists of several sections of which one is targeting people with COPD, shown in figure 1. The section targeting people with COPD aims to support self-management and includes, in addition to texts, pictures, and films also interactive components, e.g. registration of PA with person-tailored, automatised feedback. Automatised feedback in combination with step counting has been found useful to increase PA in people with COPD.[26] On the COPD Web people with COPD can gain know-how about, e.g. PA, physical training, breathing techniques, exacerbation symptoms, advice on when to contact healthcare, and how to make everyday activities less strenuous. The content refers to, and aligns with the guidelines for COPD care developed and published by the National Board of Health and Welfare in Sweden.[11]

Figure 1. A website map of the COPD Web showing the section "I have COPD".

The intervention group

Participants randomised to the intervention group will be introduced to the COPD web by a letter containing written information, the password to get access to the website and information on how to create an account. At the COPD Web, there will be an instruction movie available about how to use the COPD Web, to secure standardised instructions (Box 1).

Box 1. The content of the movie, presenting the administration of the COPD Web

1) Introduction of the website structure, the content in the main headings and functions of the website, e.g., how to enlarge or shrink the text, listen to the text, and bookmark information of particular interest.
2) Introduction to the section "Physical activity." Information about the importance of PA, and demonstration of the page for registration of PA (steps) with automated feedback.
3) Information on how to set an initial weekly step goal and instructions to insert the weekly step-count onto the page for registration of PA at the end of each week.

For peer review only

The COPD Web will be self-managed. To reduce user problems, one of the researchers (TS) will contact each participant in the first week of intervention. To test the participants' interest for and acceptability of the function of registering PA (steps) on the COPD Web, the participants will receive a pedometer with instructions on how it is used.

Prompts has shown enhanced effectiveness on limited contact interventions targeting health behaviors including PA[27] and proved to be useful also on people with COPD.[26] Throughout the intervention, participants will receive prompts via email and SMS (figure 2). The prompts will include targeted information, referral links to the COPD Web and a reminder to register counted steps to improve adherence to the intervention. There is no consensus regarding the number and frequency of prompts, but frequently delivered prompts have been recommended.[28] However too excessive appearance may decrease the desired response.[28] Consequently, the frequency of the prompts will be each week at the beginning of the intervention and decrease to biweekly (week 13 to 24) and every fourth week (week 25 to 52). In total, we will deliver 24 different prompts with predetermined content and order to each participant.

Figure 2. Distribution of prompts (SMS and email) to participants in the intervention group

The control group

The control group will, similar to the intervention group, receive a pedometer with instructions, as well as a leaflet about the importance of PA in addition to usual care. In Sweden, the majority of all people with COPD are treated at their PHCC.[11 13] The usual care at the PHCC are recommended to include, but are not restricted to, the use of long-acting anticholinergics and long-acting β2-agonists with 24 h duration and support for; smoking cessation, PA and exercise, self-management and nutrition.[11] All participants are permitted to start COPD rehabilitation or other interventions if offered by their PHCC.

Outcomes and user experience evaluation

Various methods for data collection including questionnaires, accelerometer, data from medical records (participant's latest pulmonary function test), qualitative interviews, and user data from the COPD Web will be used. Table 2 provides an overview of methods for data collection in this study.

Primary outcome measures

The primary outcome of the effect of the COPD Web is the difference in the level of PA between the intervention and control groups at the follow-ups at 3 and 12 months. The level of PA will be

For peer review only

objectively measured seven consecutive days using an accelerometer (DynaPort®, McRoberts BV, the Netherlands) and subjectively measured with indicator questions on PA from the National Board of Health and Welfare in Sweden.[29 30] Weekends and weekdays with less than eight hours of wearing time of the accelerometer and measurements with less than four valid days of measurements will be excluded.[31]The Dynaport accelerometer has been found valid and reliable when used in people with COPD.[31 32]

Secondary outcome measures

The secondary outcomes of the effect of the COPD Web are the differences between the intervention and control groups at the follow-ups at 3 and 12 months regarding participants' dyspnea; modified Medical Research Council dyspnea scale (mMRC)[33], HRQoL; Chronic Respiratory Questionnaire, self-administered (CRQ-SA)[34], and COPD-related symptoms; COPD Assessment Test (CAT).[35]

Evaluation of health economics will be done using EQ-5D[36] to estimate quality-adjusted life (QALY) gained, commonly used in economic evaluation.[37], In addition, the number of participant self-reported COPD-related healthcare contacts where a reduction in health consumption indicates a reduced economic burden.

The secondary outcomes were chosen as they cover specific aspects of the content of the COPD Web and most of them have previously been used in COPD and in a Swedish context. The range of outcomes will ensure assessment of relevant aspects of participants' symptoms and HRQoL.

Experience evaluation

For the user experience evaluation, data will be collected after three months using semi-structured individual interviews in a subgroup of participants randomised to intervention. The participants will be asked to take part in an interview at the three months follow up. The interviews will include questions regarding unexpected events or consequences of receiving the COPD Web, their use of the COPD Web, and how this use has influenced their PA behavior. Study-specific documentation and automatised data on the participants' use of the COPD Web will be collected automatically from the website, e.g., number of visits when in time they visit the site, which part of the website was used and time spent on the site.

For peer review only

Table 2 Methods for data collection

Physical objectively measured physical activity (PA) level

- Accelerometer (DynaPort, McRoberts BV (DynaPort, McRoberts BV, The Netherlands) placed on the lower back 24 hours a day over seven consecutive days.[31 32]
    - The quantity of PA will be assessed using the mean number of steps per day and the number of days per week that the participant could be considered physically active. Physically active is operationally defined as ≥5000 steps per day.
    - The Dynaport accelerometer has been found valid and reliable when used in people with COPD.[31 32]

Physical subjectively assessed PA level

- Questionnaire from the National Board of Health and Welfare.[29]
    - The time spent in physical activities such as taking a walk or working in the garden during last week is rated by choosing between pre-specified options (no time at all/30–60 min/60–90 min/9–120 min/>120 min).
    - The time spent in physical exercises such as running or doing exercise to keep fit during last week is rated by choosing between pre-specified options (no time at all/30–60 min/60–90 min/9–120 min/>120 min).
    - The categorical mode of the scale has shown low-to-moderate associations with objectively measured PA level, maximal oxygen uptake, physical performance, balance, cardiovascular biomarkers and self-rated health.[29]

Health-related quality of life (HRQoL)

- CRQ-SA The Swedish version of the self-administrated Chronic Respiratory Questionnaire.[34]
    - CRQ-SA aims to measure HRQoL in people with chronic respiratory distress. The questionnaire consists of 20 questions divided into four areas (dyspnea, fatigue, emotional function, and control) that are rated on a 7-graded Likert scale. The questions include, for example, "How often in the last two weeks have you known that you had complete control over your breathing problems?" and "In the last two weeks, how often have you known that you had low energy?".[34]
    - CRQ-SA has shown strong responsiveness to changes in HRQoL for people with COPD.[38]

COPD-related symptoms

- The questionnaire COPD Assessment Test (CAT).[35]
    - The severity of eight COPD-related symptoms (coughing, the presence of phlegm, feeling of tightness in the chest, breathlessness when walking, limitation in activities, confidence in leaving home, sleep, and energy) is rated on a six-grade scale.
    - Evaluated for internal consistency, stability over time in stable patients and ability to discriminate between stable and exacerbation patients with excellent or very good results.[35]

Dyspnea

- The questionnaire modified Medical Research Council Dyspnea Scale (mMRC).[33]
    - Perceived dyspnea is rated on a 5-graded Likert scale ranging from 0 ("I just get out of breath when I exert myself greatly" to 4 ("I get out of breath when I wash or get dressed").
    - Evaluated for categorising people with COPD in terms of disability with good results.[39]

Health economics

- Self-reported healthcare contacts related to COPD
- The questionnaire EuroQol five dimensions questionnaire(EQ-5D).[36]
    - Health status is rated on five items; three items relate to problems in mobility, self-care, and usual activities and two items cover the presence and severity of pain and anxiety/depression. Each item is rated on a three-grade scale corresponding to no problem/some or moderate problems/extreme problems.

For peer review only - http://bmjopen.bmj.com/site/about/guidelines.xhtml

- ○ Health state is rated on a scale ranging from 0 (worst imaginable health state) to 100 (best possible health state).
- ○ Evaluation of health economy will be done using EQ-5D to estimate quality-adjusted life (QALY) gained.[37] Also, the number of COPD-related health contacts and hospitalisation that occurs during the intervention will be followed and cost estimated.
- ○ EQ-5D can discriminate between groups of people with different severity of COPD.[40]

Implementation

- Implementation of the COPD Web.
  - Semi-structured interviews according to a pre-specified interview guide and user statistics from the website.
- Fidelity to the intervention.
  - Semi-structured interviews according to a pre-specified interview guide.
- Reach.
  - Study-specific documentation including the number of participants who decline to take part in the intervention. When appropriate, the reasons to decline will also be noted.
- Enablers and barriers for the use of an eHealth solution like the COPD Web
  - Semi-structured interviews according to a pre-specified interview guide.

COPD, chronic obstructive pulmonary disease.

For peer review only

Data collection, management, and analysis

Sample size calculation

The sample size was calculated with the premises that a total of 144 participants with COPD would be required to detect a mean difference of 1131 steps with a standard deviation of 2193 steps, α = 0.05, β = 0.20 (80% power), and a two-tailed test of significance[41] including an estimated dropout rate of 20%.[26]

Approximately 10-15 participants will be recruited to individual interviews to have various experiences represented. A wide distribution of age, disease severity and an equal number of women and men will be strived for.

Randomisation and masking

A permuted block design with a random block size varying from 4 to 8 in a 1:1 allocation ratio will be computer generated to randomise participants. This approach is chosen to achieve balanced and evenly distributed samples. A third party, not involved in data collection or analysis of the results will perform the randomisation and the result will be stored in sealed envelopes. Thus, the randomisation will be revealed for the researcher when the baseline registration and written informed consent are fulfilled, and the sealed envelope next in order is opened. The researcher then will send a letter containing the result of group allocation, a pedometer, a pamphlet about PA and information about when the participant will be contacted again. The members of the intervention group will, in addition, receive the material and information on how to start using the COPD Web.

Due to the character of the intervention, blinding of trial participants will not be applicable. Furthermore, as all data are self-reported, neither is blinding of outcome assessors applicable.

Data management and monitoring

To ensure confidentiality, participants with COPD will get a unique identification (ID) when included in the study. The code list linking the participants and the ID number will be kept separate from the data. Data will be analysed by ID only. All records that contain names or other personal identifiers, such as locator forms and informed consent forms, will be stored separately from study records identified by the ID number. The local database will be secured with a password-protected access system. All data will be coded and reported on a group level. Thus it will not be possible to identify specific participants in the trial. We will use two-pass verification to ensure correct data entry. No interim analyses or stopping guidelines are pre-specified. Only the researchers will have access to the final trial dataset.

Statistics and qualitative analysis

The primary analysis will be an intention-to-treat analysis (including all participants randomised). In addition, a complete case population (participants with full outcome measurements independent on adherence to intervention), and a per-protocol analysis (defined as at least one login besides creating an account on the COPD Web or answering that the SMS and email with referral links have been used at least rarely (1-3 times) at the follow-ups) will be performed. Missing data will be imputed in the intention-to-treat analysis using multiple imputation assuming data is missing at random conditional on participant severity of disease and self-reported history of exacerbations. This is because the severity of disease and history of exacerbations are known risk factors for future exacerbations and may affect adherence to PA interventions.[42]

Mixed models will be used for analysis of data with individuals at level 1 and the healthcare unit at level 2. Estimates of effect sizes will be computed using Cohen's d (d = difference in group means/error SD within). Calculated as the difference between predicted means from the final mixed-effects model for a given pair of groups divided by the estimated within-group error SD in the model with the estimated value of $2\sigma_e^2$, where σ_e^2 is the residual variance. To judge the quality of the model we, will analyse the residuals. No sub-group or adjusted analyses other than the pre-specified complete case and per-protocol analysis will be performed.

The individual interviews will be analysed using qualitative content analysis according to the procedures presented by Graneheim.[43] The interviews transcriptions will be read, coded and categorised by one researcher. Two other researchers will also read and code independently for

For peer review only

triangulation. Organisation and labeling of categories will be discussed and modified throughout the process.

Amendments

Any modifications to the protocol that may influence the conduct of the study, the potential benefit of the participant or may affect participant safety, including changes of study objectives, study design, population, sample sizes, study procedures or significant administrative aspects will require a formal amendment to the protocol. Such modifications will be agreed upon by the research group with the final decision by the principal investigator, and if needed to be approved by the local ethic committees.

Administrative changes of the protocol (e.g., minor corrections and clarifications) that do not influence how the study is conducted will be agreed upon by the research group with the final decision by the principal investigator and will be documented and presented upon publication.

Ethics approval and consent to participate

Ethical approval has been received from the Regional Ethical Review Board in Umeå, Sweden. Dnr 2018-274-31. All participants will receive brief and comprehensible oral and written information, by the Helsinki Declaration.[44] The first informed consent that confirms that contact information and latest lung function test from the potential participant will be collected by healthcare professionals and sent to the researchers. The participant will, together with the baseline registration, send the second and final informed consent to the researcher. The informed consent from operational managers will be sent and stored at the Regional Ethical Review Board in Umeå, Sweden.

Dissemination

The results of this study will be submitted for publication in peer-reviewed journals and presented at conferences both nationally and internationally as well as to included healthcare professionals, participants, and patient organisations within COPD.

Trial registration

Registration of the clinical trial before the enrolment of the first participant was performed. Date of trial initial release 2018-11-15 and published 2018-12-20. ClinicalTrials.gov identifier: NCT03746873. The recruitment began 2018-11-15 and will continue until sufficient power is reached.

For peer review only

Discussion

This study protocol presents a pragmatic randomised controlled trial with pre- and post-assessments aimed at evaluating the effect of the use of the COPD Web in people with COPD in a PHC context. The study also intends to identify enablers and barriers to use of an eHealth solution like the COPD Web to change behavior among people with COPD. Currently, despite its proven effectiveness, access to self-management interventions is limited[2 12], and alternative ways of promoting self-management for people with COPD are warranted. A recent pilot trial has shown that giving people with COPD access to the COPD Web may be an effective short-term strategy to promote self-management that increase short-term levels of PA, promote conceptual knowledge and alter disease management strategies.[21] However, these results need to be confirmed in a definitive large-scale randomised trial including both short-term and long-term evaluation.

This proposed trial will provide new knowledge to this research area by evaluating the effect of the use of an eHealth tool for increasing access to self-management strategies for people with COPD and determine its effect on clinically relevant outcomes, e.g. PA, COPD-related symptoms and dyspnea. This trial will include shorter (3 months) and longer-term perspectives (12 months) with objectively measured PA in addition to the self-reported PA that will contribute with more knowledge regarding the effect of having access to the COPD Web. PA is of utmost importance, as the level of PA is one of the strongest predictors of mortality among people with COPD.[9 10]

A user experience evaluation of the COPD Web intervention will provide novel information and understanding about enablers and barriers for the use of a web-based solution like the COPD Web to change behavior. This information will increase knowledge of how the process of receiving the intervention can be interpreted. It will also help us draw a better conclusion about if the COPD Web is accepted by the participants and about the intervention's effectiveness.

By recommendations by the pilot study, prompts will be used to encourage the use of the COPD Web during the whole intervention.[21] The reminders will provide information with referral links that will come in a predefined way. Prompts has been proven effective in other setups but there is no consensus regarding the number of prompts or frequency, especially in a longer perspective.[28] The effect of the prompts will be qualitatively evaluated through the semi-structured interviews. The evaluation will answer how the prompts were perceived and if they induced more frequent use and/or changed behavior for PA among the participants.

The use of the COPD Web will be automatically registered through the whole intervention since the participants need to log in to access the website. That measure makes it possible to analyse and answer if there is an association between the use of the COPD Web, e.g., time and number of visits and any possible effect.

For peer review only

As the study is designed as a pragmatic trial[22], the intervention will be self-managed and distance-based to maximise the clinical applicability of the findings. One concern though is that there might be participants that do not manage the instructions to create their account and learn how to use the website. However, they will be contacted at the beginning of the intervention to reduce user problems. The pragmatic approach also means that there is no selection on the number, size or location of the recruiting PHCCs. Also, the inclusion criteria are set wide with a minimised selection beyond diagnosed COPD that could enhance the recruitment rates and finally increase the clinical applicability of the findings within PHC.

One limitation is that the sample size, calculated on PA, will be large enough for evaluation of the PA but may not be large enough for all secondary outcomes or sub-group analyses. The latter much depending on the severity of symptoms among the participants.

In conclusion, the pragmatic randomised trial will provide clinically relevant information on the effect of the use of the COPD Web in people with COPD in a PHC context regarding level of PA, dyspnea, HRQoL, COPD-related symptoms and health economics in relation to healthcare use, as well as barriers and enablers for using web-based solutions such as the COPD Web.

Ethics approval and consent to participate

Regional Ethical Review Board in Umeå, Sweden.

Availability of data and materials

Not applicable.

Consent for publication

Not applicable.

Competing interests

AN reports lecture fees from AstraZeneca.

Author Contributions

TS has made direct and substantial contribution to this work by contributing to the conception and design of the study, designing and writing of the protocol. AN has made direct and substantial contribution to this work by contributing to the conception and design of the study, sample size calculation and choice of statistics, designing and writing of the protocol. SL has made direct and substantial contribution to this work in providing critical revisions that are important for the

For peer review only

intellectual content of the protocol. KW is the principal investigator and has made direct and substantial contribution to this work by providing the project idea, contributing to the conception and design of the study and by providing critical revisions that are important for the intellectual content of the protocol. All authors have approved the final version of the protocol.

Acknowledgement

Not applicable

Funding

This work was supported by The Swedish Research Council, grant number 521-2013-3503 and the Strategic Research Area – Care Science, Umeå University, Sweden, no grant number available.

References

1. World Health Organization. The top 10 causes of death.: http://www.who.int/mediacentre/factsheets/fs310/en/; [accessed 2019-03-19].
2. Jansson SA, Backman H, Stenling A, et al. Health economic costs of COPD in Sweden by disease severity - Has it changed during a ten years period? *Respir Med* 2013 doi: 10.1016/j.rmed.2013.07.012
3. Jansson SA, Backman H, Ronmark E, et al. Hospitalization Due to Co-Morbid Conditions is the Main Cost Driver Among Subjects With COPD-A Report From the Population-Based OLIN COPD Study. *Copd* 2015;12(4):381-9. doi: 10.3109/15412555.2014.974089 [published Online First: 2014/11/22]
4. Vogelmeier CF, Criner GJ, Martinez FJ, et al. Global Strategy for the Diagnosis, Management, and Prevention of Chronic Obstructive Lung Disease 2017 Report. GOLD Executive Summary. *Am J Respir Crit Care Med* 2017;195(5):557-82. doi: 10.1164/rccm.201701-0218PP [published Online First: 2017/01/28]
5. Spruit MA, Singh SJ, Garvey C, et al. An official American Thoracic Society/European Respiratory Society statement: key concepts and advances in pulmonary rehabilitation. *Am J Respir Crit Care Med* 2013;188(8):e13-64. doi: 10.1164/rccm.201309-1634ST [published Online First: 2013/10/17]
6. Effing TW, Bourbeau J, Vercoulen J, et al. Self-management programmes for COPD: moving forward. *Chronic respiratory disease* 2012;9(1):27-35. doi: 10.1177/1479972311433574
7. Apps LD, Mitchell KE, Harrison SL, et al. The development and pilot testing of the self-management programme of activity, coping and education for chronic obstructive pulmonary disease (SPACE for COPD). *International journal of chronic obstructive pulmonary disease* 2013;8:317-27. doi: 10.2147/COPD.S40414
8. Velloso M, Jardim JR. Study of energy expenditure during activities of daily living using and not using body position recommended by energy conservation techniques in patients with COPD. *Chest* 2006;130(1):126-32. doi: 10.1378/chest.130.1.126
9. Waschki B, Kirsten A, Holz O, et al. Physical activity is the strongest predictor of all-cause mortality in patients with COPD: a prospective cohort study. *Chest* 2011;140(2):331-42. doi: 10.1378/chest.10-2521 [published Online First: 2011/01/29]
10. Gimeno-Santos E, Frei A, Steurer-Stey C, et al. Determinants and outcomes of physical activity in patients with COPD: a systematic review. *Thorax* 2014;69(8):731-9. doi: 10.1136/thoraxjnl-2013-204763

For peer review only

11. Socialstyrelsen [The National Board of Health and Welfare]. Nationella riktlinjer för vård vid astma och KOL 2018. [National guidelines for asthma and COPD care] [accessed March 13, 2019].
12. Wadell K, Janaudis Ferreira T, Arne M, et al. Hospital-based pulmonary rehabilitation in patients with COPD in Sweden-A national survey. *Respir Med* 2013;107(8):1195-200. doi: 10.1016/j.rmed.2013.04.019
13. Sundh J, Lindgren H, Hasselgren M, et al. Pulmonary rehabilitation in COPD - available resources and utilization in Swedish primary and secondary care. *International journal of chronic obstructive pulmonary disease* 2017;12:1695-704. doi: 10.2147/copd.s135111 [published Online First: 2017/06/28]
14. Lundell S, Holmner A, Rehn B, et al. Telehealthcare in COPD: A systematic review and meta-analysis on physical outcomes and dyspnea. *Respir Med* 2015;109(1):11-26. doi: 10.1016/j.rmed.2014.10.008
15. Adams SG, Smith PK, Allan PF, et al. Systematic review of the chronic care model in chronic obstructive pulmonary disease prevention and management. *Archives of internal medicine* 2007;167(6):551-61. doi: 10.1001/archinte.167.6.551
16. Farmer A, Williams V, Velardo C, et al. Self-Management Support Using a Digital Health System Compared With Usual Care for Chronic Obstructive Pulmonary Disease: Randomized Controlled Trial. *J Med Internet Res* 2017;19(5):e144. doi: 10.2196/jmir.7116
17. Hanlon P, Daines L, Campbell C, et al. Telehealth Interventions to Support Self-Management of Long-Term Conditions: A Systematic Metareview of Diabetes, Heart Failure, Asthma, Chronic Obstructive Pulmonary Disease, and Cancer. *J Med Internet Res* 2017;19(5):e172. doi: 10.2196/jmir.6688 [published Online First: 2017/05/21]
18. Talboom-Kamp EP, Verdijk NA, Kasteleyn MJ, et al. The Effect of Integration of Self-Management Web Platforms on Health Status in Chronic Obstructive Pulmonary Disease Management in Primary Care (e-Vita Study): Interrupted Time Series Design. *J Med Internet Res* 2017;19(8):e291. doi: 10.2196/jmir.8262 [published Online First: 2017/08/18]
19. Tistad M, Lundell S, Wiklund M, et al. Usefulness and Relevance of an eHealth Tool in Supporting the Self-Management of Chronic Obstructive Pulmonary Disease: Explorative Qualitative Study of a Cocreative Process. *JMIR Hum Factors* 2018;5(4):e10801. doi: 10.2196/10801 [published Online First: 2018/10/29]
20. Nyberg A, Wadell K, Lindgren H, et al. Internet-based support for self-management strategies for people with COPD-protocol for a controlled pragmatic pilot trial of effectiveness and a process evaluation in primary healthcare. *BMJ open* 2017;7(7):e016851. doi: 10.1136/bmjopen-2017-016851 [published Online First: 2017/08/03]
21. Nyberg A, Tistad M, Wadell K. Can the COPD web be used to promote self-management in patients with COPD in swedish primary care: a controlled pragmatic pilot trial with 3 month- and 12 month follow-up. *Scandinavian journal of primary health care* 2019:1-14. doi: 10.1080/02813432.2019.1569415 [published Online First: 2019/02/01]
22. Zwarenstein M, Treweek S, Gagnier JJ, et al. Improving the reporting of pragmatic trials: an extension of the CONSORT statement. *BMJ (Clinical research ed)* 2008;337:a2390. doi: 10.1136/bmj.a2390 [published Online First: 2008/11/13]
23. Chan A-W, Tetzlaff JM, Altman DG, et al. SPIRIT 2013 Statement: Defining Standard Protocol Items for Clinical Trials. *Annals of Internal Medicine* 2013;158(3):200-07. doi: 10.7326/0003-4819-158-3-201302050-00583
24. Chan AW, Tetzlaff JM, Gotzsche PC, et al. SPIRIT 2013 explanation and elaboration: guidance for protocols of clinical trials. *BMJ (Clinical research ed)* 2013;346:e7586. doi: 10.1136/bmj.e7586 [published Online First: 2013/01/11]
25. Eysenbach G. CONSORT-EHEALTH: Improving and Standardizing Evaluation Reports of Web-based and Mobile Health Interventions. *J Med Internet Res* 2011;13(4):e126. doi: 10.2196/jmir.1923 [published Online First: 31.12.2011]

For peer review only

26. Demeyer H, Louvaris Z, Frei A, et al. Physical activity is increased by a 12-week semiautomated telecoaching programme in patients with COPD: a multicentre randomised controlled trial. *Thorax* 2017;72(5):415-23. doi: 10.1136/thoraxjnl-2016-209026
27. Fry JP, Neff RA. Periodic Prompts and Reminders in Health Promotion and Health Behavior Interventions: Systematic Review. *J Med Internet Res* 2009;11(2):e16. doi: 10.2196/jmir.1138 [published Online First: 14.5.2009]
28. Muench F, Baumel A. More Than a Text Message: Dismantling Digital Triggers to Curate Behavior Change in Patient-Centered Health Interventions. *J Med Internet Res* 2017;19(5):e147. doi: 10.2196/jmir.7463 [published Online First: 2017/05/28]
29. Olsson SJG, Ekblom Ö, Andersson E, et al. Categorical answer modes provide superior validity to open answers when asking for level of physical activity: A cross-sectional study. *Scandinavian Journal of Public Health* 2016;44(1):70-76. doi: 10.1177/1403494815602830
30. Socialstyrelsen [The National Board of Health and Welfare]. Nationella riktlinjer för sjukdomsförebyggande metoder 2011 Tobaksbruk, riskbruk av alkohol, otillräcklig fysisk aktivitet och ohälsosamma matvanor Stöd för styrning och ledning [Disease Prevention in the Swedish Healthcare System: Health situation, national guidelines and implementation]. (In Swedish with an English summary). Västerås, Sweden: Socialstyrelsen, 2011. .
31. Demeyer H, Burtin C, Van Remoortel H, et al. Standardizing the analysis of physical activity in patients with COPD following a pulmonary rehabilitation program. *Chest* 2014;146(2):318-27. doi: 10.1378/chest.13-1968 [published Online First: 2014/03/08]
32. Andersson M, Janson C, Emtner M. Accuracy of three activity monitors in patients with chronic obstructive pulmonary disease: a comparison with video recordings. *Copd* 2014;11(5):560-7. doi: 10.3109/15412555.2014.898033 [published Online First: 2014/04/17]
33. Mahler DA, Wells CK. Evaluation of clinical methods for rating dyspnea. *Chest* 1988;93(3):580-6. [published Online First: 1988/03/01]
34. Vernooij RW, Willson M, Gagliardi AR. Characterizing patient-oriented tools that could be packaged with guidelines to promote self-management and guideline adoption: a meta-review. *Implementation science : IS* 2016;11:52. doi: 10.1186/s13012-016-0419-1 [published Online First: 2016/04/16]
35. Jones PW, Harding G, Berry P, et al. Development and first validation of the COPD Assessment Test. *The European respiratory journal* 2009;34(3):648-54. doi: 10.1183/09031936.00102509 [published Online First: 2009/09/02]
36. Dolan P. Modeling valuations for EuroQol health states. *Medical care* 1997;35(11):1095-108. [published Online First: 1997/11/21]
37. Manca A, Hawkins N, Sculpher MJ. Estimating mean QALYs in trial-based cost-effectiveness analysis: the importance of controlling for baseline utility. *Health Economics* 2005;14(5):487-96. doi: 10.1002/hec.944
38. Puhan MA, Guyatt GH, Goldstein R, et al. Relative responsiveness of the Chronic Respiratory Questionnaire, St. Georges Respiratory Questionnaire and four other health-related quality of life instruments for patients with chronic lung disease. *Respiratory Medicine* 2007;101(2):308-16. doi: https://doi.org/10.1016/j.rmed.2006.04.023
39. Bestall JC, Paul EA, Garrod R, et al. Usefulness of the Medical Research Council (MRC) dyspnoea scale as a measure of disability in patients with chronic obstructive pulmonary disease. *Thorax* 1999;54(7):581-6. [published Online First: 1999/06/22]
40. Rutten-van Molken MP, Oostenbrink JB, Tashkin DP, et al. Does quality of life of COPD patients as measured by the generic EuroQol five-dimension questionnaire differentiate between COPD severity stages? *Chest* 2006;130(4):1117-28. doi: 10.1378/chest.130.4.1117 [published Online First: 2006/10/13]
41. Demeyer H, Burtin C, Hornikx M, et al. The Minimal Important Difference in Physical Activity in Patients with COPD. *PLOS ONE* 2016;11(4):e0154587. doi: 10.1371/journal.pone.0154587

For peer review only - http://bmjopen.bmj.com/site/about/guidelines.xhtml

42. Müllerová H, Shukla A, Hawkins A, et al. Risk factors for acute exacerbations of COPD in a primary care population: a retrospective observational cohort study. *BMJ open* 2014;4(12):e006171. doi: 10.1136/bmjopen-2014-006171
43. Graneheim UH, Lundman B. Qualitative content analysis in nursing research: concepts, procedures and measures to achieve trustworthiness. *Nurse education today* 2004;24(2):105-12. doi: 10.1016/j.nedt.2003.10.001 [published Online First: 2004/02/11]
44. World Medical A. World medical association declaration of helsinki: Ethical principles for medical research involving human subjects. *JAMA* 2013;310(20):2191-94. doi: 10.1001/jama.2013.281053

For peer review only

Table 1. Participant timeline for enrolment, the intervention and assessments

Timepoint	$t^{-1}_{\text{screening/consent}}$	t^{0}_{baseline}	t^{1}_{start}	$t^{2}_{\text{3 months}}$	$t^{3}_{\text{(interviews)}}$	$t^{4}_{\text{12 months}}$
**Enrolment**						
Eligibility screen	x					
Informed consent		x				
Allocation			x			
**Intervention**						
The COPD Web			→	→	→	→
**Assessments**						
Sociodemographics (age, sex, anthropometry, diagnosis)[1]		x		x		x
Pulmonary function[2]	x					
COPD-related symptoms[1]		x		x		x
Dyspnea[1]		x		x		x
Health-related quality of life (HRQoL)[1]		x		x		x
Time spent in physical activity and training[1]		x		x		x
Time being sedentary[1]		x		x		x
Physical activity level (accelerometer)[1]		x		x		x
Implementation [1,3]			x	x	x	x
Response to and interaction with the COPD Web[1]				x	x	x
COPD-related health care contacts[1]				x		x
Enablers and Barriers for the use of a web-based solution[1]					x	

Data collection from [1] People with COPD, [2] Medical record, [3] Statistics from the website

For peer review only

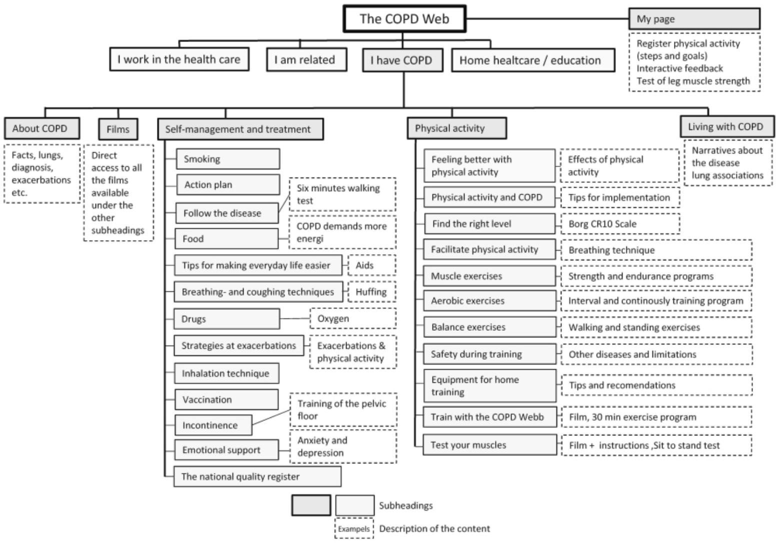

Figure 1. A website map of the COPD Web showing the section “I have COPD”.

297x209mm (300 x 300 DPI)

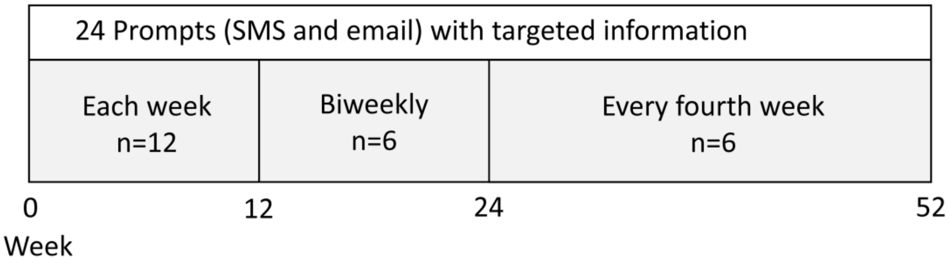

Figure 2. Distribution of prompts (SMS and email) to participants in the intervention group

183x57mm (600 x 600 DPI)

Reporting checklist for protocol of a clinical trial.

Based on the SPIRIT guidelines.

Instructions to authors

Complete this checklist by entering the page numbers from your manuscript where readers will find each of the items listed below.

Your article may not currently address all the items on the checklist. Please modify your text to include the missing information. If you are certain that an item does not apply, please write "n/a" and provide a short explanation.

Upload your completed checklist as an extra file when you submit to a journal.

In your methods section, say that you used the SPIRIT reporting guidelines, and cite them as:

Chan A-W, Tetzlaff JM, Altman DG, Laupacis A, Gøtzsche PC, Krleža-Jerić K, Hróbjartsson A, Mann H, Dickersin K, Berlin J, Doré C, Parulekar W, Summerskill W, Groves T, Schulz K, Sox H, Rockhold FW, Rennie D, Moher D. SPIRIT 2013 Statement: Defining standard protocol items for clinical trials. Ann Intern Med. 2013;158(3):200-207

		Reporting Item	Page Number
Title	#1	Descriptive title identifying the study design, population, interventions, and, if applicable, trial acronym	1
Trial registration	#2a	Trial identifier and registry name. If not yet registered, name of intended registry	2+13

For peer review only

Trial registration: data set	#2b	All items from the World Health Organization Trial Registration Data Set	n/a
Protocol version	#3	Date and version identifier	n/a
Funding	#4	Sources and types of financial, material, and other support	15
Roles and responsibilities: contributorship	#5a	Names, affiliations, and roles of protocol contributors	1+15-16
Roles and responsibilities: sponsor contact information	#5b	Name and contact information for the trial sponsor	n/a
Roles and responsibilities: sponsor and funder	#5c	Role of study sponsor and funders, if any, in study design; collection, management, analysis, and interpretation of data; writing of the report; and the decision to submit the report for publication, including whether they will have ultimate authority over any of these activities	15
Roles and responsibilities: committees	#5d	Composition, roles, and responsibilities of the coordinating centre, steering committee, endpoint adjudication committee, data management team, and other individuals or groups overseeing the trial, if applicable (see Item 21a for data monitoring committee)	n/a

For peer review only

Background and rationale	#6a	Description of research question and justification for undertaking the trial, including summary of relevant studies (published and unpublished) examining benefits and harms for each intervention	4
Background and rationale: choice of comparators	#6b	Explanation for choice of comparators	4
Objectives	#7	Specific objectives or hypotheses	5
Trial design	#8	Description of trial design including type of trial (eg, parallel group, crossover, factorial, single group), allocation ratio, and framework (eg, superiority, equivalence, non-inferiority, exploratory)	5+11
Study setting	#9	Description of study settings (eg, community clinic, academic hospital) and list of countries where data will be collected. Reference to where list of study sites can be obtained	6
Eligibility criteria	#10	Inclusion and exclusion criteria for participants. If applicable, eligibility criteria for study centres and individuals who will perform the interventions (eg, surgeons, psychotherapists)	5-6
Interventions: description	#11a	Interventions for each group with sufficient detail to allow replication, including how and when they will be administered	7-8

For peer review only

Interventions: modifications	#11b	Criteria for discontinuing or modifying allocated interventions for a given trial participant (eg, drug dose change in response to harms, participant request, or improving / worsening disease)	13
Interventions: adherance	#11c	Strategies to improve adherence to intervention protocols, and any procedures for monitoring adherence (eg, drug tablet return; laboratory tests)	8
Interventions: concomitant care	#11d	Relevant concomitant care and interventions that are permitted or prohibited during the trial	n/a
Outcomes	#12	Primary, secondary, and other outcomes, including the specific measurement variable (eg, systolic blood pressure), analysis metric (eg, change from baseline, final value, time to event), method of aggregation (eg, median, proportion), and time point for each outcome. Explanation of the clinical relevance of chosen efficacy and harm outcomes is strongly recommended	8-10
Participant timeline	#13	Time schedule of enrolment, interventions (including any run-ins and washouts), assessments, and visits for participants. A schematic diagram is highly recommended (see Figure)	6-7+20
Sample size	#14	Estimated number of participants needed to achieve study objectives and how it was determined, including clinical and statistical assumptions supporting any sample size calculations	11

For peer review only

Recruitment	#15	Strategies for achieving adequate participant enrolment to reach target sample size	6
Allocation: sequence generation	#16a	Method of generating the allocation sequence (eg, computer-generated random numbers), and list of any factors for stratification. To reduce predictability of a random sequence, details of any planned restriction (eg, blocking) should be provided in a separate document that is unavailable to those who enrol participants or assign interventions	11
Allocation concealment mechanism	#16b	Mechanism of implementing the allocation sequence (eg, central telephone; sequentially numbered, opaque, sealed envelopes), describing any steps to conceal the sequence until interventions are assigned	11
Allocation: implementation	#16c	Who will generate the allocation sequence, who will enrol participants, and who will assign participants to interventions	11
Blinding (masking)	#17a	Who will be blinded after assignment to interventions (eg, trial participants, care providers, outcome assessors, data analysts), and how	12
Blinding (masking): emergency unblinding	#17b	If blinded, circumstances under which unblinding is permissible, and procedure for revealing a participant’s allocated intervention during the trial	n/a

For peer review only

Data collection plan	#18a	Plans for assessment and collection of outcome, baseline, and other trial data, including any related processes to promote data quality (eg, duplicate measurements, training of assessors) and a description of study instruments (eg, questionnaires, laboratory tests) along with their reliability and validity, if known. Reference to where data collection forms can be found, if not in the protocol	8-11
Data collection plan: retention	#18b	Plans to promote participant retention and complete follow-up, including list of any outcome data to be collected for participants who discontinue or deviate from intervention protocols	7
Data management	#19	Plans for data entry, coding, security, and storage, including any related processes to promote data quality (eg, double data entry; range checks for data values). Reference to where details of data management procedures can be found, if not in the protocol	12
Statistics: outcomes	#20a	Statistical methods for analysing primary and secondary outcomes. Reference to where other details of the statistical analysis plan can be found, if not in the protocol	12-13
Statistics: additional analyses	#20b	Methods for any additional analyses (eg, subgroup and adjusted analyses)	12

For peer review only

Statistics: analysis population and missing data	#20c	Definition of analysis population relating to protocol non-adherence (eg, as randomised analysis), and any statistical methods to handle missing data (eg, multiple imputation)	12
Data monitoring: formal committee	#21a	Composition of data monitoring committee (DMC); summary of its role and reporting structure; statement of whether it is independent from the sponsor and competing interests; and reference to where further details about its charter can be found, if not in the protocol. Alternatively, an explanation of why a DMC is not needed	n/a
Data monitoring: interim analysis	#21b	Description of any interim analyses and stopping guidelines, including who will have access to these interim results and make the final decision to terminate the trial	12
Harms	#22	Plans for collecting, assessing, reporting, and managing solicited and spontaneously reported adverse events and other unintended effects of trial interventions or trial conduct	9
Auditing	#23	Frequency and procedures for auditing trial conduct, if any, and whether the process will be independent from investigators and the sponsor	n/a
Research ethics approval	#24	Plans for seeking research ethics committee / institutional review board (REC / IRB) approval	2+13+15

For peer review only

Protocol amendments	#25	Plans for communicating important protocol modifications (eg, changes to eligibility criteria, outcomes, analyses) to relevant parties (eg, investigators, REC / IRBs, trial participants, trial registries, journals, regulators)	13
Consent or assent	#26a	Who will obtain informed consent or assent from potential trial participants or authorised surrogates, and how (see Item 32)	6+12-13
Consent or assent: ancillary studies	#26b	Additional consent provisions for collection and use of participant data and biological specimens in ancillary studies, if applicable	n/a
Confidentiality	#27	How personal information about potential and enrolled participants will be collected, shared, and maintained in order to protect confidentiality before, during, and after the trial	12
Declaration of interests	#28	Financial and other competing interests for principal investigators for the overall trial and each study site	14
Data access	#29	Statement of who will have access to the final trial dataset, and disclosure of contractual agreements that limit such access for investigators	12
Ancillary and post trial care	#30	Provisions, if any, for ancillary and post-trial care, and for compensation to those who suffer harm from trial participation	n/a

For peer review only

Dissemination policy: trial results	#31a	Plans for investigators and sponsor to communicate trial results to participants, healthcare professionals, the public, and other relevant groups (eg, via publication, reporting in results databases, or other data sharing arrangements), including any publication restrictions	13
Dissemination policy: authorship	#31b	Authorship eligibility guidelines and any intended use of professional writers	n/a
Dissemination policy: reproducible research	#31c	Plans, if any, for granting public access to the full protocol, participant-level dataset, and statistical code	n/a
Informed consent materials	#32	Model consent form and other related documentation given to participants and authorised surrogates	n/a
Biological specimens	#33	Plans for collection, laboratory evaluation, and storage of biological specimens for genetic or molecular analysis in the current trial and for future use in ancillary studies, if applicable	n/a

The SPIRIT checklist is distributed under the terms of the Creative Commons Attribution License CC-BY-ND 3.0. This checklist was completed on 29. March 2019 using https://www.goodreports.org/, a tool made by the EQUATOR Network in collaboration with Penelope.ai

For peer review only

BMJ Open

Web-based support for self-management strategies versus usual care for people with COPD in primary healthcare: a protocol for a randomised, 12 months, parallel-group pragmatic trial.

Journal:	*BMJ Open*
Manuscript ID	bmjopen-2019-030788.R1
Article Type:	Protocol
Date Submitted by the Author:	22-Aug-2019
Complete List of Authors:	Stenlund, Tobias; Umea University Department of Community Medicine and Rehabilitation, Physiotherapy<br>Nyberg, André; Umea University Department of Community Medicine and Rehabilitation, Physiotherapy<br>Lundell, Sara; Umea University Department of Community Medicine and Rehabilitation, Physiotherapy<br>Wadell, Karin; Umea University Department of Community Medicine and Rehabilitation, Physiotherapy
**Primary Subject Heading**:	Respiratory medicine
Secondary Subject Heading:	Health informatics
Keywords:	Pulmonary Disease, Chronic obstructive, eHealth, self-management strategies, PRIMARY CARE

SCHOLARONE™
Manuscripts

Web-based support for self-management strategies versus usual care for people with COPD in primary healthcare: a protocol for a randomised, 12 months, parallel-group pragmatic trial.

AUTHORS

Tobias Stenlund, PhD, Dept. of Community Medicine and Rehabilitation, Physiotherapy, Umeå University, Umeå, 90187 Sweden. Tel.: +46907868040. E-mail: tobias.stenlund@umu.se

André Nyberg, Associate Professor, Dept. of Community Medicine and Rehabilitation, Physiotherapy, Umeå University, Umeå, 90187 Sweden. E-mail: andre.nyberg@umu.se

Sara Lundell, PhD, Dept. of Community Medicine and Rehabilitation, Physiotherapy, Umeå University, Umeå, 90187 Sweden. E-mail: sara.lundell@umu.se

Karin Wadell, Professor, Dept. of Community Medicine and Rehabilitation, Physiotherapy, Umeå University, Umeå, 90187 Sweden. E-mail: karin.wadell@umu.se

Corresponding author: PhD Tobias Stenlund Dept. of Community Medicine and Rehabilitation, Physiotherapy, Umeå University, Umeå, 90187 Sweden. Tel.: +46907868040. E-mail: tobias.stenlund@umu.se

Keywords: Pulmonary Disease, Chronic obstructive; eHealth; primary care; self-management strategies

Word Count: 3976

For peer review only

ABSTRACT

Introduction

The use of adequate self-management strategies for people with chronic obstructive pulmonary disease (COPD) may increase level of physical activity (PA), improve health-related quality of life (HRQoL) and reduce healthcare use. Whether web-based support in addition to prompts (e-mail and SMS) could be used to promote self-management strategies to facilitate behavior change in people with COPD is not clear. This clinical trial aims to generate evidence on the effect of a web-based solution, the COPD Web, in a cohort of people with COPD in a primary healthcare context.

Methods and analysis

The overall design is a pragmatic randomised controlled trial with pre- and post-assessments (3 and 12 months) and a implementation and user experience evaluation. People with a diagnosis of COPD, treated in primary healthcare will be eligible for the study. A total of 144 participants will be enrolled by healthcare professionals at included primary healthcare units and, after fulfilled baseline assessments, randomised to either control or intervention group. All participants will receive usual care, a pedometer and a leaflet about the importance of PA. Participants in the intervention will, in addition, get access to the COPD Web, an interactive self-managed website that aims to support people with COPD in self-management strategies. They will also continuously get support from prompts with focus on behaviour change.

The effect on participants' PA, dyspnea, COPD related symptoms, HRQoL, and health economics will be assessed using accelerometer and questionnaires. To identify enablers and barriers for the use of web-based support to change behavior, semistructured interviews will be conducted in a subgroup of participants at the 3 months follow-up.

Ethics and dissemination

Ethical approval has been received from the Regional Ethical Review Board in Umeå, Sweden. Dnr 2018-274-31. Findings will be presented at conferences, submitted for publication in peer-reviewed journals and presented to the involved healthcare professionals, participants and patient organisations.

Trial registration number

ClinicalTrials.gov: NCT03746873

For peer review only

Article Summary

Strengths and limitations of this study

- Physical activity level will be objectively measured and bring the field forward regarding knowledge about both short- and long-term effects of using web-based support.
- Information on how and how much the participants have used the COPD Web will automatically be collected and analysed throughout the full intervention period, which will increase the understanding of the link between use of the COPD Web and the possible effects.
- The pragmatic design with generous inclusion criteria and many recruiting primary healthcare units could enhance external validity.
- Prompts will be sent continuously as a reminder and strategy to encourage greater exposures to the COPD Web.
- One limitation is that the sample size is large enough for analysing the effect on physical activity level but may not be large enough for all secondary outcomes.

For peer review only

Introduction

Background and rationale

Chronic obstructive pulmonary disease (COPD) is a chronic and disabling disease with substantial morbidity and mortality. The disease has a steady increase in prevalence and is now the third leading cause of death worldwide.[1] The high prevalence places a considerable burden on the healthcare system with a total yearly cost of COPD in Sweden estimated to 13.9 billion SEK.[2] The mean annual total costs for each person with COPD is 67% higher compared to a person without COPD.[3]

The symptom burden of the disease; respiratory symptoms as progressive dyspnea, fatigue, impaired physical performance, decreased level of physical activity (PA) and health-related quality of life (HRQoL)[4] is not only a consequence of the underlying condition, but depend also on the individuals' adaptation to the illness and their ability to manage their disease.[5 6] Self-management strategies, including strategies to promote change in health behaviour by increasing the individual's knowledge and skills and their confidence in successfully managing their disease, is therefore now an essential part of COPD management.[5] This have shown to reduce breathlessness and impact of COPD in daily life, increase physical performance, level of PA, HRQoL, adherence to medication, as well as improve time to recovery after acute exacerbations and reduce overall health-related costs.[5 7 8] An increased level of PA is of utmost importance and something to promote[9] since PA has been shown to be decreased early in the disease progression[10] and degree of PA is considered the strongest predictor of all-cause mortality in people with COPD.[11 12]

Despite that treatment guidelines and literature strongly supports that non-pharmacological treatment (i.e., education, self-management strategies, exercise training)[13] should be provided, the vast majority of people with COPD are still excluded from these activities.[14 15] Web-based solutions are promising means of delivering health service, and may increase level of PA[16 17] as well as reduced use of health services.[18] However, studies evaluating whether web-based support could be used to promote self-management strategies to support increased PA in people with COPD are contradictory. One showed no effect on PA while other studies showed improved PA[19-21] but that the improvement may not be sustained over a long duration.[21]

The COPD Web is a web-based solution, developed by our research group in co-creation with people with COPD, their relatives, healthcare professionals in primary healthcare (PHC) and researchers.[22] In a pilot study on 83 people with COPD[23 24] promising results with increased self-reported level of PA were shown. To know whether this is true also for a larger COPD population an adequately powered randomised controlled trial with short and long-term evaluation is needed.

For peer review only

Objectives

The main aim is to generate evidence on the effect of the COPD Web in a cohort of people with COPD, currently enrolled for usual care within the PHC context in Sweden. This is of importance, as the vast majority of people with COPD are treated within PHC.[13 15] The specific aims are to evaluate the short and long-term effect of the use of the COPD Web in an adequately powered group of people with COPD in PHC context, regarding i) level of PA; ii) dyspnea iii) HRQoL, iv) COPD related symptoms, v) health economics in relation to healthcare use; and vi) to identify enablers and barriers for the the use of web-based support with the COPD Web in order to change behavior.

We hypothesise that access and use of the COPD Web, in comparison to usual care, will:

i) increase level of objectively measured PA in people with COPD,
ii) decrease dyspnea,
iii) increase disease-specific HRQoL,
iv) decrease number of and/or severity of COPD-related symptoms, and
v) decrease number of COPD-related healthcare contacts in PHC.

Methods and analysis

Trial design

The design is a pragmatic randomised controlled trial with pre- and post-assessments (3 and 12 months) in addition to a user experience and implementation evaluation. The user experience and implementation evaluation is a necessary complement to understand more about enablers and barriers for behavior change using web-based support. The study is designed as a pragmatic trial[25] meaning that healthcare professionals, primarily COPD nurses, are involved in recruiting participants, the access to the intervention (COPD Web) is given by the researchers, but the intervention itself only uses self-instructional material and prompts (SMS and email). This design aims to minimise the effort from healthcare professionals and increase the possibility of self-management for people with COPD to improve the applicability of the findings to other healthcare settings. The protocol complies with the SPIRIT (Standard Protocol Items: Recommendations for Interventional Trials) recommendations for protocol reporting[26 27] checklist (Additional file 1) and the study will be reported according to CONSORT (Consolidated Standards of Reporting Trials) guidelines for pragmatic trials[25] and eHealth.[28]

Patient and Public Involvement (PPI)

We did not directly include PPI in this study, but our research group in co-creation with PPI developed the COPD Web used in the study.

For peer review only

Participants and intervention

Study settings

PHC units from different County Councils in Sweden, will constitute the study sites. The number of units are not limited; consequently, more units may be included during the study. At present 25 units are included, 13 of them situated in urban areas and 12 located in smaller cities or rural areas. The number of enrolled citizens at the included units range between 5,700 and 20,300 citizens. One unit has no enrolled citizens but act as a rehabilitation unit that treats patients with a referral from other PHC units. We will include both publicy funded PHC units and private alternatives.

Eligibility criteria

The trial will be conducted from 15 November 2018 until 144 participants are included. All people with a diagnosis of COPD (ICD-10:J44:9) who visit involved PHCCs due to their COPD will be eligible for inclusion in the study if they 1) can read and understand Swedish, 2) have a smartphone, tablet or computer with access to internet, 3) don't have dementia or other psychiatric condition that can prevent understanding of the intervention, 4) don't have severe comorbidity that can be considered as the contributing factor for limitation in PA, and 5) don't already use the COPD Web. In the case of exacerbation, the participant has to wait six weeks from the start of pharmacological treatment, before being eligible to the study.

Participant timeline

The recruitment begins at included PHC units. To facilitate the recruitment of participants, the number of included units will not be restricted to nor the units size, location, how they are funded or the type of care and rehabilitation that the unit offers. Written consent from the operational manager has to be fulfilled before recruitment can start.

To increase the possibility of recruiting participants, the number of exclusion criteria are kept to a minimum. The recruitment will take place during the participant's regular visits to the PHC unit where healthcare professionals will give information about the study. People with COPD interested in participation will have their contact information and results from latest pulmonary function test (if older than six months, a new pulmonary function test will be performed) sent to the research group as displayed in table 1. A researcher (TS) will after verbal agreement send informed consent form, questionnaires and activity monitor for baseline assessment to the participants' homes. When the written informed consent and the baseline assessment is fulfilled, the participants' are included and randomised to either the control or intervention group. Follow-up measurements with questionnaires and activity monitor will be conducted at 3 and 12 months after inclusion. A semi-

For peer review only

structured interview will be done after the 3 months follow-up among a convenient sample of the intervention group.

The participants will be contacted by phone before every assessment to ensure a suitable date for the activity monitoring. In case of non-response after any evaluation the participant will be reminded by phone or/and email weekly. These precautions will be made to maintain the participant in the study and increase the number of complete follow-ups.

Intervention

The COPD Web consists of several sections of which one is targeting people with COPD, shown in figure 1. The section targeting people with COPD aims to support self-management and includes, in addition to texts, pictures, and films also interactive components, e.g. registration of PA with person-tailored, automatised feedback. Automatised feedback in combination with step counting has been found useful to increase PA in people with COPD.[29] On the website, people with COPD can gain know-how about, e.g. PA, physical training, breathing techniques, exacerbation symptoms, advice on when to contact healthcare, and how to make everyday activities less strenuous. The content refers to, and aligns with the guidelines for COPD care developed and published by the National Board of Health and Welfare in Sweden.[13]

Figure 1. A website map of the COPD Web showing the section "I have COPD".

The intervention group

Participants randomised to the intervention group will be introduced to the COPD web by a letter containing written information, the password to get access to the website and information on how to create an account. To secure standardised instructions there will be an instruction movie available on the website, (Box 1).

Box 1. The content of the movie, presenting the administration of the COPD Web

1) Introduction of the website structure, the content in the main headings and functions of the website, e.g., how to enlarge or shrink the text, listen to the text, and bookmark information of particular interest.
2) Introduction to the section "Physical activity." Information about the importance of PA, and demonstration of the page for registration of PA (steps) with automated feedback.
3) Information on how to set an initial weekly step goal and instructions to insert the weekly step-count onto the page for registration of PA at the end of each week.

For peer review only

The COPD Web will be self-managed. To reduce user problems, one of the researchers (TS) will contact each participant in the first week of intervention. To test the participants' interest for and acceptability of the function of registering PA (steps) on the website, the participants will receive a pedometer with instructions on how it is used.

Throughout the intervention, participants will receive prompts via email and SMS (figure 2). The prompts will include targeted information, referral links to the COPD Web and a reminder to register counted steps to improve adherence to the intervention. Prompts has shown enhanced effectiveness on limited contact interventions targeting health behaviors including PA[30] and proved to be useful also on people with COPD[29] though there is no consensus regarding the number and frequency of prompts. Frequently delivered prompts have been recommended however too excessive appearance may decrease the desired response.[31] Consequently, the frequency of the prompts will be each week at the beginning of the intervention and decrease to biweekly (week 13 to 24) and every fourth week (week 25 to 52). In total, we will deliver 24 different prompts with predetermined content and order to each participant.

Figure 2. Distribution of prompts (SMS and email) to participants in the intervention group

The control group

The control group will, similar to the intervention group, receive a pedometer with instructions, as well as a leaflet about the importance of PA in addition to usual care. In Sweden, the majority of all people with COPD are treated within PHC.[13 15] Usual care within PHC are recommended to include, but are not restricted to, use of long-acting anticholinergics and long-acting β2-agonists with 24 h duration and support for; smoking cessation, PA and exercise, self-management and nutrition.[13] All participants are permitted to start COPD rehabilitation or other interventions if offered at their PHC unit.

Outcomes and evaluation

Various methods for data collection including questionnaires, accelerometer, data from medical records (participant's latest pulmonary function test), qualitative interviews, and user data from the COPD Web will be used. Table 2 provides an overview of methods for data collection in this study.

Primary outcome measures

The primary outcome of the effect of the COPD Web is the difference in level of PA between intervention and control groups at follow-ups (3 and 12 months). Level of PA will be objectively measured seven consecutive days using an accelerometer (DynaPort®, McRoberts BV, the

For peer review only

Netherlands) and subjectively measured with indicator questions on PA from the National Board of Health and Welfare in Sweden.[32 33] Weekends and weekdays with less than eight hours of wearing time of the accelerometer and measurements with less than four valid days of measurements will be excluded.[34]The Dynaport accelerometer has been found valid and reliable when used in people with COPD.[34 35]

Secondary outcome measures

The secondary outcomes of the effect of the COPD Web are the differences between the intervention and control groups at the follow-ups at 3 and 12 months regarding participants' dyspnea; modified Medical Research Council dyspnea scale (mMRC)[36], HRQoL; Chronic Respiratory Questionnaire, self-administered (CRQ-SA)[37], and COPD-related symptoms; COPD Assessment Test (CAT).[38] Evaluation of health economics will be done using EQ-5D[39] to estimate quality-adjusted life (QALY) gained, commonly used in economic evaluation.[40] In addition, the number of participant self-reported COPD-related healthcare contacts will be evaluated where a reduction in health consumption indicates a reduced economic burden. Secondary outcomes were chosen according to results in the pilot study and since they cover specific aspects of the content of the COPD Web. Most of them have previously been used in COPD and in a Swedish context.

User experience and implementation evaluation

For user experience evaluation, data will be collected after 3 months using semi-structured individual interviews in a subgroup of participants randomised to intervention. The participants will be asked to take part in an interview at 3 months follow-up. The interviews will include questions regarding unexpected events or consequences of receiving the COPD Web, their use of the website, and how this use has influenced their PA behavior. Study-specific documentation and automatised data on the participants' use of the COPD Web will be collected automatically from the website, e.g., number of visits, pages was used and time spent on the website. This will ad valuable information to the exeperince valuation but also make it possible to evaluate the fidelity to the intervention. In order to evaluate the implemtention and who is reached and not reached study-specific documentation including the number of participants who decline to take part in the intervention or drop outs will be noted. In addition, when appropriate, will the reasons to decline also be noted. All participants will also with the questionnaires answer study-specific questions regarding other ongoing or started interventions, hospitalisations or exacerbations that could affect the results.

For peer review only

Table 2 Methods for data collection

Physical objectively measured physical activity (PA) level

- Accelerometer (DynaPort, McRoberts BV (DynaPort®, McRoberts BV, The Netherlands) placed on the lower back 24 hours a day over seven consecutive days.[34 35]
  - The quantity of PA will be assessed using the mean number of steps per day and the number of days per week that the participant could be considered physically active. Physically active is operationally defined as ≥5000 steps per day.
  - The Dynaport accelerometer has been found valid and reliable when used in people with COPD.[34 35]

Physical subjectively assessed PA level

- Questionnaire from the National Board of Health and Welfare.[33]
  - The time spent in physical activities such as taking a walk or working in the garden during last week is rated by choosing between pre-specified options (no time at all/30–60 min/60–90 min/9–120 min/>120 min).
  - The time spent in physical exercises such as running or doing exercise to keep fit during last week is rated by choosing between pre-specified options (no time at all/30–60 min/60–90 min/9–120 min/>120 min).
  - The categorical mode of the scale has shown low-to-moderate associations with objectively measured PA level, maximal oxygen uptake, physical performance, balance, cardiovascular biomarkers and self-rated health.[32]

Health-related quality of life (HRQoL)

- CRQ-SA The Swedish version of the self-administrated Chronic Respiratory Questionnaire.[37]
  - CRQ-SA aims to measure HRQoL in people with chronic respiratory distress. The questionnaire consists of 20 questions divided into four areas (dyspnea, fatigue, emotional function, and control) that are rated on a 7-graded Likert scale. The questions include, for example, "How often in the last two weeks have you known that you had complete control over your breathing problems?" and "In the last two weeks, how often have you known that you had low energy?".[37]
  - CRQ-SA has shown strong responsiveness to changes in HRQoL for people with COPD.[41]

COPD-related symptoms

- The questionnaire COPD Assessment Test (CAT).[38]
  - The severity of eight COPD-related symptoms (coughing, the presence of phlegm, feeling of tightness in the chest, breathlessness when walking, limitation in activities, confidence in leaving home, sleep, and energy) is rated on a six-grade scale.
  - Evaluated for internal consistency, stability over time in stable patients and ability to discriminate between stable and exacerbation patients with excellent or very good results.[38]

Dyspnea

- The questionnaire modified Medical Research Council Dyspnea Scale (mMRC).[36]
  - Perceived dyspnea is rated on a 5-graded Likert scale ranging from 0 ("I just get out of breath when I exert myself greatly" to 4 ("I get out of breath when I wash or get dressed").
  - Evaluated for categorising people with COPD in terms of disability with good results.[42]

Health economics

- Self-reported healthcare contacts related to COPD
- The questionnaire EuroQol five dimensions questionnaire(EQ-5D).[39]
  - Health status is rated on five items; three items relate to problems in mobility, self-care, and usual activities and two items cover the presence and severity of pain and anxiety/depression. Each item is rated on a three-grade scale corresponding to no problem/some or moderate problems/extreme problems.

For peer review only

- General health is rated on a scale ranging from 0 (worst imaginable health state) to 100 (best possible health state).
- Evaluation of health economy will be done using EQ-5D to estimate quality-adjusted life (QALY) gained.[40] Also, the number of COPD-related health contacts and hospitalisation that occurs during the intervention will be followed and cost estimated.
- EQ-5D can discriminate between groups of people with different severity of COPD.[43]

Implementation

- Implementation of the COPD Web.
  - Semi-structured interviews will be performed according to a pre-specified interview guide and user statistics from the website will be analysed.
- Fidelity to the intervention.
  - Semi-structured interviews will be performed according to a pre-specified interview guide.
- Reach.
  - Study-specific documentation including the number of participants who decline to take part in the intervention will be analysed. When appropriate, the reasons to decline will be noted.
- Enablers and barriers for the use of web-based support like the COPD Web
  - Semi-structured interviews will be performed according to a pre-specified interview guide and analysed.

COPD, chronic obstructive pulmonary disease.

Data collection, management, and analysis

Sample size calculation

The sample size was calculated with the premises that a total of 144 participants with COPD would be required to detect a mean difference of 1131 steps with a standard deviation of 2193 steps[44], α = 0.05, β = 0.20 (80% power), and a two-tailed test of significance including an estimated dropout rate of 20%.[29] Approximately 10-15 participants will be recruited to individual interviews to have various experiences represented. A wide distribution of age, disease severity and an equal number of women and men will be strived for.

Randomisation and masking

A permuted block design with a random block size varying from 4 to 8 in a 1:1 allocation ratio will be computer generated to randomise participants. This approach is chosen to achieve balanced and evenly distributed samples. A third party, not involved in data collection or analysis of the results, will perform the randomisation and the result will be stored in sealed envelopes. Thus, the randomisation will be revealed for the researcher when the baseline registration and written informed consent are fulfilled, and the sealed envelope next in order is opened. The researcher then will send a letter containing the result of group allocation, a pedometer, a pamphlet about PA and information about when the participant will be contacted again. The members of the intervention group will, in addition, receive the material and information on how to start using the COPD Web.

For peer review only

Due to the character of the intervention, blinding of trial participants will not be applicable. Furthermore, as all data are self-reported, neither is blinding of outcome assessors applicable.

Data management and monitoring

To ensure confidentiality, participants with COPD will get a unique identification (ID) when included in the study. The code list linking participants and ID number will be kept separate from the data. Data will be analysed by ID only. All records that contain names or other personal identifiers, such as locator forms and informed consent forms, will be stored separately from study records identified by the ID number. The local database will be secured with a password-protected access system. All data will be coded and reported on group level. Thus it will not be possible to identify specific participants in the trial. We will use two-pass verification to ensure correct data entry. No interim analyses or stopping guidelines are pre-specified. Only the researchers will have access to the final trial dataset.

Statistics and qualitative analysis

The primary analysis will be an intention-to-treat analysis (including all participants randomised). In addition, a complete case population (participants with full outcome measurements independent on adherence to intervention), and a per-protocol analysis (defined as at least one login besides creating an account on the COPD Web or answering that the SMS and email with referral links have been used at least rarely (1-3 times) at the follow-ups) will be performed. Missing data will be imputed in the intention-to-treat analysis using multiple imputation assuming data is missing at random conditional on participants' severity of disease and self-reported history of exacerbations. This is because severity of disease and history of exacerbations are known risk factors for future exacerbations and may affect adherence to PA interventions.[45]

The difference in primary outcome between intervention and control group will be estimated using multilevel mixed effects models with subjects at level 1 and PHC units at level 2. PHC units and subjects will be modelled as random effects while group (intervention group vs control group), time and group*time interaction as fixed effects. Estimates of effect sizes will be computed using Cohen's d (d = difference in group means/error SD within). Calculated as the difference between predicted means from the final mixed-effects model for a given pair of groups divided by the estimated within-group error SD in the model with the estimated value of $2\sigma_e^2$, where σ_e^2 is the residual variance. To judge the quality of the model we, will analyse the residuals. No sub-group or adjusted analyses other than the pre-specified complete case and per-protocol analysis will be performed.

The individual interviews will be analysed using qualitative content analysis according to the procedures presented by Graneheim.[46] The interviews transcriptions will be read, coded and categorised by one researcher. Two other researchers will also read and code independently for

For peer review only

triangulation. Organisation and labeling of categories will be discussed and modified throughout the process.

Amendments

Any modifications to the protocol that may influence the conduct of the study, the potential benefit of the participant or may affect participant safety, including changes of study objectives, study design, population, sample sizes, study procedures or significant administrative aspects will require a formal amendment to the protocol. Such modifications will be agreed upon by the research group with the final decision by the principal investigator, and if needed to be approved by the local ethic committees.

Administrative changes of the protocol (e.g., minor corrections and clarifications) that do not influence how the study is conducted will be agreed upon by the research group with the final decision by the principal investigator and will be documented and presented upon publication.

Ethics approval and consent to participate

Ethical approval has been received from the Regional Ethical Review Board in Umeå, Sweden. Dnr 2018-274-31. All participants will receive brief, comprehensible oral and written information, by the Helsinki Declaration.[47] A first informed consent confirms that contact information and latest pulmonary function test from the potential participant can be collected by healthcare professionals and sent to the researchers. The participant will, together with the baseline assessment, send a second and final informed consent to the researcher. The informed consent from operational managers will be sent and stored at the Regional Ethical Review Board in Umeå, Sweden.

Dissemination

The results of this study will be submitted for publication in peer-reviewed journals and presented at conferences both nationally and internationally as well as to included healthcare professionals, participants, and patient organisations for people with COPD.

Trial registration

Registration of the clinical trial before the enrolment of the first participant was performed. Date of trial initial release 2018-11-15 and published 2018-12-20. ClinicalTrials.gov identifier: NCT03746873. The recruitment began 2018-11-15 and will continue until sufficient power is reached.

For peer review only

Discussion

This study protocol presents a pragmatic randomised controlled trial with pre- and post-assessments aimed at evaluating the effect of the use of the COPD Web for people with COPD in a PHC context. The study also intends to evaluate implementation and to identify enablers and barriers to use of web-based support to change behavior among people with COPD. Currently, despite its proven effectiveness, access to self-management interventions is limited[2 14], and alternative ways of promoting self-management for people with COPD are warranted. A recent pilot trial has shown that giving people with COPD access to the COPD Web may be an effective short-term strategy to promote self-management that increase levels of PA, promote conceptual knowledge and alter disease management strategies.[24] However, these results need to be confirmed in a definitive large-scale randomised trial including both short- and long-term evaluation.

This proposed trial will provide new knowledge to this research area by evaluating the effect of the use of web-based support for increasing access to self-management strategies for people with COPD and determine its effect on clinically relevant outcomes. This trial will include short- (3 months) and long-term perspectives (12 months) with objectively measured PA in addition to the self-reported PA that will contribute with more knowledge regarding the effect of having access to the COPD Web. PA is of utmost importance, as the level of PA is one of the strongest predictors of mortality among people with COPD.[11 12]

A user experience and implementation evaluation of the intervention will provide novel information and understanding about enablers and barriers for the use of web-based support to change behavior. This information will increase knowledge of how the process of receiving the intervention can be interpreted. It will also help us draw a better conclusions regarding acceptance, fidelity and implementation of the COPD Web.

Guided by the pilot study, prompts will be used to encourage the use of the website during the intervention period.[24] The reminders will provide information with referral links that will appear in a predefined way. Prompts have been proven effective in other setups but there is no consensus regarding the number of prompts or frequency, especially in a longer perspective.[31] The effect of the prompts will be qualitatively evaluated through the semi-structured interviews. The evaluation will answer how the prompts were perceived and if they induced more frequent use and/or changed behaviour regarding PA among the participants. The use of the COPD Web will be automatically registered through the whole intervention since the participants need to log in to access the website. That measure makes it possible to analyse the fidelity to the intervention and answer if there is an

For peer review only

association between the use of the COPD Web, e.g., time and number of visits and any possible effect.

As the study is designed as a pragmatic trial[25], the intervention will be self-managed and distance-based to maximise the clinical applicability of the findings. One concern is that there might be participants who do not manage the instructions to create their account and learn how to use the website. However, they will be contacted at the beginning of the intervention to reduce user problems. The pragmatic approach also means that there is no selection on the number, size or location of the recruiting PHC units. Also, the inclusion criteria are set wide with a minimised selection beyond diagnosed COPD that could enhance the recruitment rates and finally increase the clinical applicability of the findings within PHC. One limitation is that the sample size, calculated on PA, will be large enough for evaluation of the PA but may not be powered enough for all secondary outcome or sub-group analyses. The latter much depending on the severity of symptoms among the participants.

In conclusion, this pragmatic randomised trial will provide clinically relevant information on the effect of the use of the COPD Web in people with COPD in a PHC context regarding level of PA, dyspnea, HRQoL, COPD-related symptoms and health economics in relation to healthcare use, as well as barriers and enablers for using web-based support with solutions such as the COPD Web.

Ethics approval and consent to participate

Regional Ethical Review Board in Umeå, Sweden.

Availability of data and materials

Not applicable.

Consent for publication

Not applicable.

Competing interests

AN reports lecture fees from AstraZeneca.

Author Contributions

TS has made direct and substantial contribution to this work by contributing to the conception and design of the study, designing and writing of the protocol. AN has made direct and substantial

For peer review only

contribution to this work by contributing to the conception and design of the study, sample size calculation and choice of statistics, designing and writing of the protocol. SL has made direct and substantial contribution to this work in providing critical revisions that are important for the intellectual content of the protocol. KW is the principal investigator and has made direct and substantial contribution to this work by providing the project idea, contributing to the conception and design of the study and by providing critical revisions that are important for the intellectual content of the protocol. All authors have approved the final version of the protocol.

Acknowledgement

Not applicable

Funding

This work was supported by The Swedish Research Council, grant number 521-2013-3503 and the Strategic Research Area – Care Science, Umeå University, Sweden, no grant number available.

References

1. World Health Organization. The top 10 causes of death.: http://www.who.int/mediacentre/factsheets/fs310/en/; [accessed 2019-03-19].
2. Jansson SA, Backman H, Stenling A, et al. Health economic costs of COPD in Sweden by disease severity - Has it changed during a ten years period? *Respir Med* 2013 doi: 10.1016/j.rmed.2013.07.012
3. Jansson SA, Backman H, Ronmark E, et al. Hospitalization Due to Co-Morbid Conditions is the Main Cost Driver Among Subjects With COPD-A Report From the Population-Based OLIN COPD Study. *Copd* 2015;12(4):381-9. doi: 10.3109/15412555.2014.974089 [published Online First: 2014/11/22]
4. Vogelmeier CF, Criner GJ, Martinez FJ, et al. Global Strategy for the Diagnosis, Management, and Prevention of Chronic Obstructive Lung Disease 2017 Report. GOLD Executive Summary. *Am J Respir Crit Care Med* 2017;195(5):557-82. doi: 10.1164/rccm.201701-0218PP [published Online First: 2017/01/28]
5. Spruit MA, Singh SJ, Garvey C, et al. An official American Thoracic Society/European Respiratory Society statement: key concepts and advances in pulmonary rehabilitation. *Am J Respir Crit Care Med* 2013;188(8):e13-64. doi: 10.1164/rccm.201309-1634ST [published Online First: 2013/10/17]
6. Effing TW, Bourbeau J, Vercoulen J, et al. Self-management programmes for COPD: moving forward. *Chronic respiratory disease* 2012;9(1):27-35. doi: 10.1177/1479972311433574
7. Apps LD, Mitchell KE, Harrison SL, et al. The development and pilot testing of the self-management programme of activity, coping and education for chronic obstructive pulmonary disease (SPACE for COPD). *International journal of chronic obstructive pulmonary disease* 2013;8:317-27. doi: 10.2147/COPD.S40414
8. Velloso M, Jardim JR. Study of energy expenditure during activities of daily living using and not using body position recommended by energy conservation techniques in patients with COPD. *Chest* 2006;130(1):126-32. doi: 10.1378/chest.130.1.126

For peer review only

9. Garcia-Aymerich J, Pitta F. Promoting Regular Physical Activity in Pulmonary Rehabilitation. *Clinics in Chest Medicine* 2014;35(2):363-68. doi: 10.1016/j.ccm.2014.02.011
10. Troosters T, Sciurba F, Battaglia S, et al. Physical inactivity in patients with COPD, a controlled multi-center pilot-study. *Respir Med* 2010;104(7):1005-11. doi: 10.1016/j.rmed.2010.01.012 [published Online First: 2010/02/20]
11. Waschki B, Kirsten A, Holz O, et al. Physical activity is the strongest predictor of all-cause mortality in patients with COPD: a prospective cohort study. *Chest* 2011;140(2):331-42. doi: 10.1378/chest.10-2521 [published Online First: 2011/01/29]
12. Gimeno-Santos E, Frei A, Steurer-Stey C, et al. Determinants and outcomes of physical activity in patients with COPD: a systematic review. *Thorax* 2014;69(8):731-9. doi: 10.1136/thoraxjnl-2013-204763
13. Socialstyrelsen [The National Board of Health and Welfare]. Nationella riktlinjer för vård vid astma och KOL 2018. [National guidelines for asthma and COPD care] [accessed March 13, 2019].
14. Wadell K, Janaudis Ferreira T, Arne M, et al. Hospital-based pulmonary rehabilitation in patients with COPD in Sweden-A national survey. *Respir Med* 2013;107(8):1195-200. doi: 10.1016/j.rmed.2013.04.019
15. Sundh J, Lindgren H, Hasselgren M, et al. Pulmonary rehabilitation in COPD - available resources and utilization in Swedish primary and secondary care. *International journal of chronic obstructive pulmonary disease* 2017;12:1695-704. doi: 10.2147/copd.s135111 [published Online First: 2017/06/28]
16. Lundell S, Holmner A, Rehn B, et al. Telehealthcare in COPD: A systematic review and meta-analysis on physical outcomes and dyspnea. *Respir Med* 2015;109(1):11-26. doi: 10.1016/j.rmed.2014.10.008
17. Loeckx M, Rabinovich RA, Demeyer H, et al. Smartphone-Based Physical Activity Telecoaching in Chronic Obstructive Pulmonary Disease: Mixed-Methods Study on Patient Experiences and Lessons for Implementation. *JMIR mHealth and uHealth* 2018;6(12):e200. doi: 10.2196/mhealth.9774
18. Adams SG, Smith PK, Allan PF, et al. Systematic review of the chronic care model in chronic obstructive pulmonary disease prevention and management. *Archives of internal medicine* 2007;167(6):551-61. doi: 10.1001/archinte.167.6.551
19. Moy ML, Collins RJ, Martinez CH, et al. An Internet-Mediated Pedometer-Based Program Improves Health-Related Quality-of-Life Domains and Daily Step Counts in COPD: A Randomized Controlled Trial. *Chest* 2015;148(1):128-37. doi: 10.1378/chest.14-1466 [published Online First: 2015/03/27]
20. Wan ES, Kantorowski A, Homsy D, et al. Promoting physical activity in COPD: Insights from a randomized trial of a web-based intervention and pedometer use. *Respir Med* 2017;130:102-10. doi: 10.1016/j.rmed.2017.07.057 [published Online First: 2017/12/06]
21. McCabe C, McCann M, Brady AM. Computer and mobile technology interventions for self-management in chronic obstructive pulmonary disease. *Cochrane Database of Systematic Reviews* 2017(5) doi: 10.1002/14651858.CD011425.pub2
22. Tistad M, Lundell S, Wiklund M, et al. Usefulness and Relevance of an eHealth Tool in Supporting the Self-Management of Chronic Obstructive Pulmonary Disease: Explorative Qualitative Study of a Cocreative Process. *JMIR Hum Factors* 2018;5(4):e10801. doi: 10.2196/10801 [published Online First: 2018/10/29]
23. Nyberg A, Wadell K, Lindgren H, et al. Internet-based support for self-management strategies for people with COPD-protocol for a controlled pragmatic pilot trial of effectiveness and a process evaluation in primary healthcare. *BMJ open* 2017;7(7):e016851. doi: 10.1136/bmjopen-2017-016851 [published Online First: 2017/08/03]
24. Nyberg A, Tistad M, Wadell K. Can the COPD web be used to promote self-management in patients with COPD in swedish primary care: a controlled pragmatic pilot trial with 3 month-

For peer review only

and 12 month follow-up. *Scandinavian journal of primary health care* 2019:1-14. doi: 10.1080/02813432.2019.1569415 [published Online First: 2019/02/01]
25. Zwarenstein M, Treweek S, Gagnier JJ, et al. Improving the reporting of pragmatic trials: an extension of the CONSORT statement. *BMJ (Clinical research ed)* 2008;337:a2390. doi: 10.1136/bmj.a2390 [published Online First: 2008/11/13]
26. Chan A-W, Tetzlaff JM, Altman DG, et al. SPIRIT 2013 Statement: Defining Standard Protocol Items for Clinical Trials. *Annals of Internal Medicine* 2013;158(3):200-07. doi: 10.7326/0003-4819-158-3-201302050-00583
27. Chan AW, Tetzlaff JM, Gotzsche PC, et al. SPIRIT 2013 explanation and elaboration: guidance for protocols of clinical trials. *BMJ (Clinical research ed)* 2013;346:e7586. doi: 10.1136/bmj.e7586 [published Online First: 2013/01/11]
28. Eysenbach G. CONSORT-EHEALTH: Improving and Standardizing Evaluation Reports of Web-based and Mobile Health Interventions. *J Med Internet Res* 2011;13(4):e126. doi: 10.2196/jmir.1923 [published Online First: 31.12.2011]
29. Demeyer H, Louvaris Z, Frei A, et al. Physical activity is increased by a 12-week semiautomated telecoaching programme in patients with COPD: a multicentre randomised controlled trial. *Thorax* 2017;72(5):415-23. doi: 10.1136/thoraxjnl-2016-209026
30. Fry JP, Neff RA. Periodic Prompts and Reminders in Health Promotion and Health Behavior Interventions: Systematic Review. *J Med Internet Res* 2009;11(2):e16. doi: 10.2196/jmir.1138 [published Online First: 14.5.2009]
31. Muench F, Baumel A. More Than a Text Message: Dismantling Digital Triggers to Curate Behavior Change in Patient-Centered Health Interventions. *J Med Internet Res* 2017;19(5):e147. doi: 10.2196/jmir.7463 [published Online First: 2017/05/28]
32. Olsson SJG, Ekblom Ö, Andersson E, et al. Categorical answer modes provide superior validity to open answers when asking for level of physical activity: A cross-sectional study. *Scandinavian Journal of Public Health* 2016;44(1):70-76. doi: 10.1177/1403494815602830
33. Socialstyrelsen [The National Board of Health and Welfare]. Nationella riktlinjer för sjukdomsförebyggande metoder 2011 Tobaksbruk, riskbruk av alkohol, otillräcklig fysisk aktivitet och ohälsosamma matvanor Stöd för styrning och ledning [Disease Prevention in the Swedish Healthcare System: Health situation, national guidelines and implementation]. (In Swedish with an English summary). Västerås, Sweden: Socialstyrelsen, 2011. .
34. Demeyer H, Burtin C, Van Remoortel H, et al. Standardizing the analysis of physical activity in patients with COPD following a pulmonary rehabilitation program. *Chest* 2014;146(2):318-27. doi: 10.1378/chest.13-1968 [published Online First: 2014/03/08]
35. Andersson M, Janson C, Emtner M. Accuracy of three activity monitors in patients with chronic obstructive pulmonary disease: a comparison with video recordings. *Copd* 2014;11(5):560-7. doi: 10.3109/15412555.2014.898033 [published Online First: 2014/04/17]
36. Mahler DA, Wells CK. Evaluation of clinical methods for rating dyspnea. *Chest* 1988;93(3):580-6. [published Online First: 1988/03/01]
37. Vernooij RW, Willson M, Gagliardi AR. Characterizing patient-oriented tools that could be packaged with guidelines to promote self-management and guideline adoption: a meta-review. *Implementation science : IS* 2016;11:52. doi: 10.1186/s13012-016-0419-1 [published Online First: 2016/04/16]
38. Jones PW, Harding G, Berry P, et al. Development and first validation of the COPD Assessment Test. *The European respiratory journal* 2009;34(3):648-54. doi: 10.1183/09031936.00102509 [published Online First: 2009/09/02]
39. Dolan P. Modeling valuations for EuroQol health states. *Medical care* 1997;35(11):1095-108. [published Online First: 1997/11/21]
40. Manca A, Hawkins N, Sculpher MJ. Estimating mean QALYs in trial-based cost-effectiveness analysis: the importance of controlling for baseline utility. *Health Economics* 2005;14(5):487-96. doi: 10.1002/hec.944

For peer review only

41. Puhan MA, Guyatt GH, Goldstein R, et al. Relative responsiveness of the Chronic Respiratory Questionnaire, St. Georges Respiratory Questionnaire and four other health-related quality of life instruments for patients with chronic lung disease. *Respiratory Medicine* 2007;101(2):308-16. doi: https://doi.org/10.1016/j.rmed.2006.04.023
42. Bestall JC, Paul EA, Garrod R, et al. Usefulness of the Medical Research Council (MRC) dyspnoea scale as a measure of disability in patients with chronic obstructive pulmonary disease. *Thorax* 1999;54(7):581-6. [published Online First: 1999/06/22]
43. Rutten-van Molken MP, Oostenbrink JB, Tashkin DP, et al. Does quality of life of COPD patients as measured by the generic EuroQol five-dimension questionnaire differentiate between COPD severity stages? *Chest* 2006;130(4):1117-28. doi: 10.1378/chest.130.4.1117 [published Online First: 2006/10/13]
44. Demeyer H, Burtin C, Hornikx M, et al. The Minimal Important Difference in Physical Activity in Patients with COPD. *PLOS ONE* 2016;11(4):e0154587. doi: 10.1371/journal.pone.0154587
45. Müllerová H, Shukla A, Hawkins A, et al. Risk factors for acute exacerbations of COPD in a primary care population: a retrospective observational cohort study. *BMJ open* 2014;4(12):e006171. doi: 10.1136/bmjopen-2014-006171
46. Graneheim UH, Lundman B. Qualitative content analysis in nursing research: concepts, procedures and measures to achieve trustworthiness. *Nurse education today* 2004;24(2):105-12. doi: 10.1016/j.nedt.2003.10.001 [published Online First: 2004/02/11]
47. World Medical A. World medical association declaration of helsinki: Ethical principles for medical research involving human subjects. *JAMA* 2013;310(20):2191-94. doi: 10.1001/jama.2013.281053

For peer review only

Table 1. Participant timeline for enrolment, the intervention and assessments

Timepoint	t^{-1} screening/consent	t^{0} baseline	t^{1} start	t^{2} 3 months	t^{3} (interviews)	t^{4} 12 months
**Enrolment**						
Eligibility screen	x					
Informed consent		x				
Allocation			x			
**Intervention**						
The COPD Web			→	→	→	→
**Assessments**						
Sociodemographics (age, sex, anthropometry, diagnosis)[1]		x		x		x
Pulmonary function[2]	x					
COPD-related symptoms[1]		x		x		x
Dyspnea[1]		x		x		x
Health-related quality of life (HRQoL)[1]		x		x		x
Time spent in physical activity and training[1]		x		x		x
Time being sedentary[1]		x		x		x
Physical activity level (accelerometer)[1]		x		x		x
Implementation [1,3]			x	x	x	x
Response to and interaction with the COPD Web[1]				x	x	x
COPD-related health care contacts[1]				x		x
Enablers and Barriers for the use of a web-based solution[1]					x	

Data collection from [1] People with COPD, [2] Medical record, [3] Statistics from the website

For peer review only - http://bmjopen.bmj.com/site/about/guidelines.xhtml

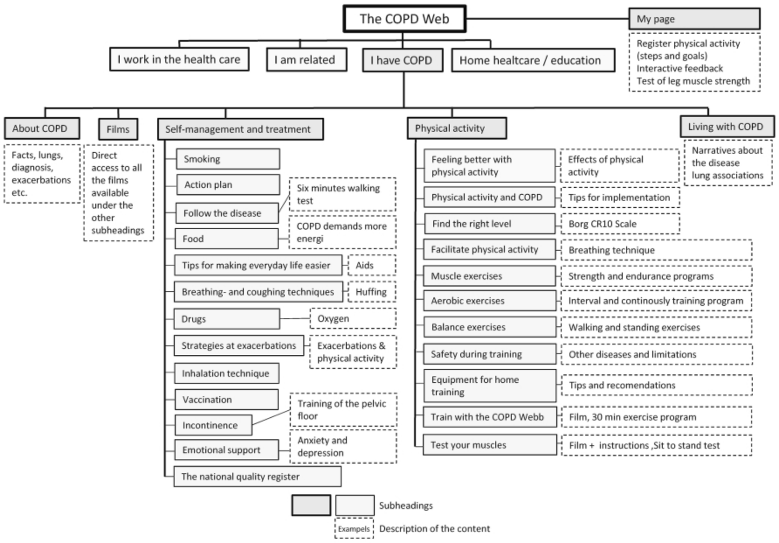

Figure 1. A website map of the COPD Web showing the section “I have COPD”.

297x209mm (300 x 300 DPI)

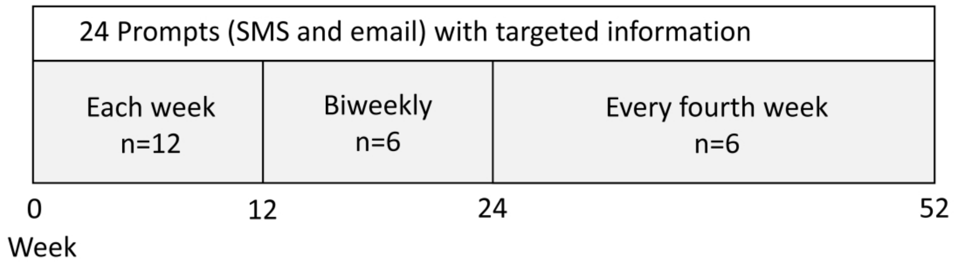

Figure 2. Distribution of prompts (SMS and email) to participants in the intervention group

183x57mm (600 x 600 DPI)

Reporting checklist for protocol of a clinical trial.

Based on the SPIRIT guidelines.

Instructions to authors

Complete this checklist by entering the page numbers from your manuscript where readers will find each of the items listed below.

Your article may not currently address all the items on the checklist. Please modify your text to include the missing information. If you are certain that an item does not apply, please write "n/a" and provide a short explanation.

Upload your completed checklist as an extra file when you submit to a journal.

In your methods section, say that you used the SPIRIT reporting guidelines, and cite them as:

Chan A-W, Tetzlaff JM, Altman DG, Laupacis A, Gøtzsche PC, Krleža-Jerić K, Hróbjartsson A, Mann H, Dickersin K, Berlin J, Doré C, Parulekar W, Summerskill W, Groves T, Schulz K, Sox H, Rockhold FW, Rennie D, Moher D. SPIRIT 2013 Statement: Defining standard protocol items for clinical trials. Ann Intern Med. 2013;158(3):200-207

		Reporting Item	Page Number
Title	#1	Descriptive title identifying the study design, population, interventions, and, if applicable, trial acronym	1
Trial registration	#2a	Trial identifier and registry name. If not yet registered, name of intended registry	2+13
Trial registration: data set	#2b	All items from the World Health Organization Trial Registration Data Set	n/a
Protocol version	#3	Date and version identifier	n/a
Funding	#4	Sources and types of financial, material, and other support	15
Roles and responsibilities: contributorship	#5a	Names, affiliations, and roles of protocol contributors	1+15-16

For peer review only

Roles and responsibilities: sponsor contact information	#5b	Name and contact information for the trial sponsor	n/a
Roles and responsibilities: sponsor and funder	#5c	Role of study sponsor and funders, if any, in study design; collection, management, analysis, and interpretation of data; writing of the report; and the decision to submit the report for publication, including whether they will have ultimate authority over any of these activities	15
Roles and responsibilities: committees	#5d	Composition, roles, and responsibilities of the coordinating centre, steering committee, endpoint adjudication committee, data management team, and other individuals or groups overseeing the trial, if applicable (see Item 21a for data monitoring committee)	n/a
Background and rationale	#6a	Description of research question and justification for undertaking the trial, including summary of relevant studies (published and unpublished) examining benefits and harms for each intervention	4
Background and rationale: choice of comparators	#6b	Explanation for choice of comparators	4
Objectives	#7	Specific objectives or hypotheses	5
Trial design	#8	Description of trial design including type of trial (eg, parallel group, crossover, factorial, single group), allocation ratio, and framework (eg, superiority, equivalence, non-inferiority, exploratory)	5+11
Study setting	#9	Description of study settings (eg, community clinic, academic hospital) and list of countries where data will be collected. Reference to where list of study sites can be obtained	6
Eligibility criteria	#10	Inclusion and exclusion criteria for participants. If applicable, eligibility criteria for study centres and individuals who will perform the interventions (eg, surgeons, psychotherapists)	5-6

For peer review only

Interventions: description	#11a	Interventions for each group with sufficient detail to allow replication, including how and when they will be administered	7-8
Interventions: modifications	#11b	Criteria for discontinuing or modifying allocated interventions for a given trial participant (eg, drug dose change in response to harms, participant request, or improving / worsening disease)	13
Interventions: adherance	#11c	Strategies to improve adherence to intervention protocols, and any procedures for monitoring adherence (eg, drug tablet return; laboratory tests)	8
Interventions: concomitant care	#11d	Relevant concomitant care and interventions that are permitted or prohibited during the trial	n/a
Outcomes	#12	Primary, secondary, and other outcomes, including the specific measurement variable (eg, systolic blood pressure), analysis metric (eg, change from baseline, final value, time to event), method of aggregation (eg, median, proportion), and time point for each outcome. Explanation of the clinical relevance of chosen efficacy and harm outcomes is strongly recommended	8-10
Participant timeline	#13	Time schedule of enrolment, interventions (including any run-ins and washouts), assessments, and visits for participants. A schematic diagram is highly recommended (see Figure)	6-7+20
Sample size	#14	Estimated number of participants needed to achieve study objectives and how it was determined, including clinical and statistical assumptions supporting any sample size calculations	11
Recruitment	#15	Strategies for achieving adequate participant enrolment to reach target sample size	6
Allocation: sequence generation	#16a	Method of generating the allocation sequence (eg, computer-generated random numbers), and list of any factors for stratification. To reduce predictability of a random sequence, details of any planned restriction (eg, blocking) should be provided in a separate document that	11

For peer review only

		is unavailable to those who enrol participants or assign interventions	
Allocation concealment mechanism	#16b	Mechanism of implementing the allocation sequence (eg, central telephone; sequentially numbered, opaque, sealed envelopes), describing any steps to conceal the sequence until interventions are assigned	11
Allocation: implementation	#16c	Who will generate the allocation sequence, who will enrol participants, and who will assign participants to interventions	11
Blinding (masking)	#17a	Who will be blinded after assignment to interventions (eg, trial participants, care providers, outcome assessors, data analysts), and how	12
Blinding (masking): emergency unblinding	#17b	If blinded, circumstances under which unblinding is permissible, and procedure for revealing a participant's allocated intervention during the trial	n/a
Data collection plan	#18a	Plans for assessment and collection of outcome, baseline, and other trial data, including any related processes to promote data quality (eg, duplicate measurements, training of assessors) and a description of study instruments (eg, questionnaires, laboratory tests) along with their reliability and validity, if known. Reference to where data collection forms can be found, if not in the protocol	8-11
Data collection plan: retention	#18b	Plans to promote participant retention and complete follow-up, including list of any outcome data to be collected for participants who discontinue or deviate from intervention protocols	7
Data management	#19	Plans for data entry, coding, security, and storage, including any related processes to promote data quality (eg, double data entry; range checks for data values). Reference to where details of data management procedures can be found, if not in the protocol	12
Statistics: outcomes	#20a	Statistical methods for analysing primary and secondary outcomes. Reference to where other details of the statistical analysis plan can be found, if not in the protocol	12-13

For peer review only

Statistics: additional analyses	#20b	Methods for any additional analyses (eg, subgroup and adjusted analyses)	12
Statistics: analysis population and missing data	#20c	Definition of analysis population relating to protocol non-adherence (eg, as randomised analysis), and any statistical methods to handle missing data (eg, multiple imputation)	12
Data monitoring: formal committee	#21a	Composition of data monitoring committee (DMC); summary of its role and reporting structure; statement of whether it is independent from the sponsor and competing interests; and reference to where further details about its charter can be found, if not in the protocol. Alternatively, an explanation of why a DMC is not needed	n/a
Data monitoring: interim analysis	#21b	Description of any interim analyses and stopping guidelines, including who will have access to these interim results and make the final decision to terminate the trial	12
Harms	#22	Plans for collecting, assessing, reporting, and managing solicited and spontaneously reported adverse events and other unintended effects of trial interventions or trial conduct	9
Auditing	#23	Frequency and procedures for auditing trial conduct, if any, and whether the process will be independent from investigators and the sponsor	n/a
Research ethics approval	#24	Plans for seeking research ethics committee / institutional review board (REC / IRB) approval	2+13+15
Protocol amendments	#25	Plans for communicating important protocol modifications (eg, changes to eligibility criteria, outcomes, analyses) to relevant parties (eg, investigators, REC / IRBs, trial participants, trial registries, journals, regulators)	13
Consent or assent	#26a	Who will obtain informed consent or assent from potential trial participants or authorised surrogates, and how (see Item 32)	6+12-13

For peer review only

Consent or assent: ancillary studies	#26b	Additional consent provisions for collection and use of participant data and biological specimens in ancillary studies, if applicable	n/a
Confidentiality	#27	How personal information about potential and enrolled participants will be collected, shared, and maintained in order to protect confidentiality before, during, and after the trial	12
Declaration of interests	#28	Financial and other competing interests for principal investigators for the overall trial and each study site	14
Data access	#29	Statement of who will have access to the final trial dataset, and disclosure of contractual agreements that limit such access for investigators	12
Ancillary and post trial care	#30	Provisions, if any, for ancillary and post-trial care, and for compensation to those who suffer harm from trial participation	n/a
Dissemination policy: trial results	#31a	Plans for investigators and sponsor to communicate trial results to participants, healthcare professionals, the public, and other relevant groups (eg, via publication, reporting in results databases, or other data sharing arrangements), including any publication restrictions	13
Dissemination policy: authorship	#31b	Authorship eligibility guidelines and any intended use of professional writers	n/a
Dissemination policy: reproducible research	#31c	Plans, if any, for granting public access to the full protocol, participant-level dataset, and statistical code	n/a
Informed consent materials	#32	Model consent form and other related documentation given to participants and authorised surrogates	n/a
Biological specimens	#33	Plans for collection, laboratory evaluation, and storage of biological specimens for genetic or molecular analysis in the current trial and for future use in ancillary studies, if applicable	n/a

The SPIRIT checklist is distributed under the terms of the Creative Commons Attribution License CC-BY-ND 3.0. This checklist was completed on 29. March 2019 using https://www.goodreports.org/, a tool made by the EQUATOR Network in collaboration with Penelope.ai

For peer review only

BMJ Open

Web-based support for self-management strategies versus usual care for people with COPD in primary healthcare: a protocol for a randomised, 12 months, parallel-group pragmatic trial.

Journal:	*BMJ Open*
Manuscript ID	bmjopen-2019-030788.R2
Article Type:	Protocol
Date Submitted by the Author:	06-Sep-2019
Complete List of Authors:	Stenlund, Tobias; Umea University Department of Community Medicine and Rehabilitation, Physiotherapy<br>Nyberg, André; Umea University Department of Community Medicine and Rehabilitation, Physiotherapy<br>Lundell, Sara; Umea University Department of Community Medicine and Rehabilitation, Physiotherapy<br>Wadell, Karin; Umea University Department of Community Medicine and Rehabilitation, Physiotherapy
**Primary Subject Heading**:	Respiratory medicine
Secondary Subject Heading:	Health informatics
Keywords:	Pulmonary Disease, Chronic obstructive, eHealth, self-management strategies, PRIMARY CARE

SCHOLARONE™
Manuscripts

Web-based support for self-management strategies versus usual care for people with COPD in primary healthcare: a protocol for a randomised, 12 months, parallel-group pragmatic trial.

AUTHORS

Tobias Stenlund, PhD, Dept. of Community Medicine and Rehabilitation, Physiotherapy, Umeå University, Umeå, 90187 Sweden. Tel.: +46907868040. E-mail: tobias.stenlund@umu.se

André Nyberg, Associate Professor, Dept. of Community Medicine and Rehabilitation, Physiotherapy, Umeå University, Umeå, 90187 Sweden. E-mail: andre.nyberg@umu.se

Sara Lundell, PhD, Dept. of Community Medicine and Rehabilitation, Physiotherapy, Umeå University, Umeå, 90187 Sweden. E-mail: sara.lundell@umu.se

Karin Wadell, Professor, Dept. of Community Medicine and Rehabilitation, Physiotherapy, Umeå University, Umeå, 90187 Sweden. E-mail: karin.wadell@umu.se

Corresponding author: PhD Tobias Stenlund Dept. of Community Medicine and Rehabilitation, Physiotherapy, Umeå University, Umeå, 90187 Sweden. Tel.: +46907868040. E-mail: tobias.stenlund@umu.se

Keywords: Pulmonary Disease, Chronic obstructive; eHealth; primary care; self-management strategies

Word Count: 3984

For peer review only

ABSTRACT

Introduction

The use of adequate self-management strategies for people with chronic obstructive pulmonary disease (COPD) may increase the level of physical activity (PA), improve health-related quality of life (HRQoL) and reduce healthcare use. Whether web-based support in addition to prompts (e-mail and SMS) could be used to promote self-management strategies to facilitate behaviour change in people with COPD is not clear. This clinical trial aims to generate evidence on the effect of a web-based solution, the COPD Web, in a cohort of people with COPD in a primary healthcare context.

Methods and analysis

The overall design is a pragmatic randomised controlled trial with pre- and post-assessments (3 and 12 months) and an implementation and user experience evaluation. People with a diagnosis of COPD, treated in primary healthcare will be eligible for the study. A total of 144 participants will be enrolled by healthcare professionals at included primary healthcare units and, after fulfilled baseline assessments, randomised to either control or intervention group. All participants will receive usual care, a pedometer, and a leaflet about the importance of PA. Participants in the intervention will, in addition, get access to the COPD Web, an interactive self-managed website that aims to support people with COPD in self-management strategies. They will also continuously get support from prompts with a focus on behaviour change.

The effect on participants' PA, dyspnea, COPD related symptoms, HRQoL, and health economics will be assessed using accelerometer and questionnaires. To identify enablers and barriers for the use of web-based support to change behaviour, semistructured interviews will be conducted in a subgroup of participants at the 3 months follow-up.

Ethics and dissemination

Ethical approval has been received from the Regional Ethical Review Board in Umeå, Sweden. Dnr 2018-274-31. Findings will be presented at conferences, submitted for publication in peer-reviewed journals and presented to the involved healthcare professionals, participants, and patient organisations.

Trial registration number

ClinicalTrials.gov: NCT03746873

For peer review only

Article Summary

Strengths and limitations of this study

- Physical activity level will be objectively measured and bring the field forward regarding knowledge about both short- and long-term effects of using web-based support.
- Information on how and how much the participants have used the COPD Web will automatically be collected and analysed throughout the full intervention period, which will increase the understanding of the link between the use of the COPD Web and the possible effects.
- The pragmatic design with generous inclusion criteria and many recruiting primary healthcare units could enhance external validity.
- Prompts will be sent continuously as a reminder and strategy to encourage greater exposures to the COPD Web.
- One limitation is that the sample size is large enough for analysing the effect on physical activity level but may not be large enough for all secondary outcomes.

For peer review only

Introduction

Background and rationale

Chronic obstructive pulmonary disease (COPD) is a chronic and disabling disease with substantial morbidity and mortality. The disease has a steady increase in prevalence and is now the third leading cause of death worldwide.[1] The high prevalence places a considerable burden on the healthcare system with a total yearly cost of COPD in Sweden estimated to 13.9 billion SEK.[2] The mean annual total costs for each person with COPD is 67% higher compared to a person without COPD.[3]

The symptom burden of the disease; respiratory symptoms as progressive dyspnea, fatigue, impaired physical performance, decreased level of physical activity (PA) and health-related quality of life (HRQoL)[4] is not only a consequence of the underlying condition, but depend also on the individuals' adaptation to the illness and their ability to manage their disease.[5 6] Self-management strategies, including strategies to promote change in health behaviour by increasing the individual's knowledge and skills and their confidence in successfully managing their disease, is therefore now an essential part of COPD management.[5] This have shown to reduce breathlessness and impact of COPD in daily life, increase physical performance, level of PA, HRQoL, adherence to medication, as well as improve time to recovery after acute exacerbations and reduce overall health-related costs.[5 7 8] An increased level of PA is of utmost importance and something to promote[9] since PA has been shown to be decreased early in the disease progression[10] and degree of PA is considered the strongest predictor of all-cause mortality in people with COPD.[11 12]

Despite that treatment guidelines and literature strongly supports that non-pharmacological treatment (i.e., education, self-management strategies, exercise training)[13] should be provided, the vast majority of people with COPD are still excluded from these activities.[14 15] Web-based solutions are promising means of delivering health service, and may increase level of PA[16 17] as well as reduced use of health services.[18] However, studies evaluating whether web-based support could be used to promote self-management strategies to support increased PA in people with COPD are contradictory. One showed no effect on PA while other studies showed improved PA[19-21] but that the improvement may not be sustained over a long duration.[21]

The COPD Web is a web-based solution, developed by our research group in co-creation with people with COPD, their relatives, healthcare professionals in primary healthcare (PHC) and researchers.[22] In a pilot study on 83 people with COPD[23 24] promising results with an increased self-reported level of PA were shown. To know whether this is true also for a larger COPD population, an adequately powered randomised controlled trial with short and long-term evaluation is needed.

For peer review only

Objectives

The main aim is to generate evidence on the effect of the COPD Web in a cohort of people with COPD, currently enrolled for usual care within the PHC context in Sweden. This is of importance, as the vast majority of people with COPD are treated within PHC.[13 15] The specific aims are to evaluate the short and long-term effect of the use of the COPD Web in an adequately powered group of people with COPD in PHC context, regarding i) level of PA; ii) dyspnea iii) HRQoL, iv) COPD related symptoms, v) health economics in relation to healthcare use; and vi) to identify enablers and barriers for the use of web-based support with the COPD Web in order to change behaviour.

We hypothesise that access and use of the COPD Web, in comparison to usual care, will:

i) increase level of objectively measured PA in people with COPD,
ii) decrease dyspnea,
iii) increase disease-specific HRQoL,
iv) decrease the number of and/or severity of COPD-related symptoms, and
v) decrease the number of COPD-related healthcare contacts in PHC.

Methods and analysis

Trial design

The design is a pragmatic randomised controlled trial with pre- and post-assessments (3 and 12 months) in addition to user experience and implementation evaluation. The user experience and implementation evaluation is a necessary complement to understand more about enablers and barriers for behaviour change using web-based support. The study is designed as a pragmatic trial[25] meaning that healthcare professionals, primarily COPD nurses, are involved in recruiting participants, the access to the intervention (COPD Web) is given by the researchers, but the intervention itself only uses self-instructional material and prompts (SMS and email). This design aims to minimise the effort from healthcare professionals and increase the possibility of self-management for people with COPD to improve the applicability of the findings to other healthcare settings. The protocol complies with the SPIRIT (Standard Protocol Items: Recommendations for Interventional Trials) recommendations for protocol reporting[26 27] (Additional file 1) and the study will be reported according to CONSORT (Consolidated Standards of Reporting Trials) guidelines for pragmatic trials[25] and eHealth.[28]

Patient and Public Involvement (PPI)

We did not directly include PPI in this study, but our research group in co-creation with PPI developed the COPD Web used in the study.

For peer review only

Participants and intervention

Study settings

PHC units from different County Councils in Sweden will constitute the study sites. The number of units is not limited; consequently, more units may be included during the study. At present 25 units are included, 13 of them situated in urban areas and 12 located in smaller cities or rural areas. The number of enrolled citizens at the included units range between 5,700 and 20,300 citizens. One unit has no enrolled citizens but acts as a rehabilitation unit that treats patients with a referral from other PHC units. We will include both publicly funded PHC units and private alternatives.

Eligibility criteria

The trial will be conducted from 15 November 2018 until 144 participants are included. All people with a diagnosis of COPD (ICD-10:J44:9) who visit involved PHCCs due to their COPD will be eligible for inclusion in the study if they 1) can read and understand Swedish, 2) have a smartphone, tablet or computer with access to internet, 3) don't have dementia or other psychiatric condition that can prevent understanding of the intervention, 4) don't have severe comorbidity that can be considered as the contributing factor for limitation in PA, and 5) don't already use the COPD Web. In the case of exacerbation, the participant has to wait six weeks from the start of pharmacological treatment, before being eligible to the study.

Participant timeline

The recruitment begins at included PHC units. To facilitate the recruitment of participants, the number of included units will not be restricted to nor the units size, location, how they are funded or the type of care and rehabilitation that the unit offers. Written consent from the operational manager has to be fulfilled before recruitment can start.

To increase the possibility of recruiting participants, the number of exclusion criteria are kept to a minimum. The recruitment will take place during the participant's regular visits to the PHC unit where healthcare professionals will give information about the study. People with COPD interested in participation will have their contact information and results from latest pulmonary function test (if older than six months, a new pulmonary function test will be performed) sent to the research group as displayed in table 1. A researcher (TS) will after verbal agreement send informed consent form, questionnaires, and activity monitor for baseline assessment to the participants' homes. When the written informed consent and the baseline assessment is fulfilled, the participants' are included and randomised to either the control or intervention group. Follow-up measurements with questionnaires and activity monitor will be conducted at 3 and 12 months after inclusion. A semi-

For peer review only

structured interview will be done after the 3 months follow-up among a convenient sample of the intervention group.

The participants will be contacted by phone before every assessment to ensure a suitable date for the activity monitoring. In case of non-response after any evaluation, the participant will be reminded by phone or/and email weekly. These precautions will be made to maintain the participant in the study and increase the number of complete follow-ups.

Intervention

The COPD Web consists of several sections of which one is targeting people with COPD, shown in figure 1. The section targeting people with COPD aims to support self-management and includes, in addition to texts, pictures, and films, also interactive components, e.g. registration of PA with person-tailored, automatised feedback. Automatised feedback in combination with step counting has been found useful to increase PA in people with COPD.[29] On the website, people with COPD can gain know-how about, e.g. PA, physical training, breathing techniques, exacerbation symptoms, advice on when to contact healthcare, and how to make everyday activities less strenuous. The content refers to and aligns with the guidelines for COPD care developed and published by the National Board of Health and Welfare in Sweden.[13]

Figure 1. A website map of the COPD Web showing the section "I have COPD".

The intervention group

Participants randomised to the intervention group will be introduced to the COPD Web by a letter containing written information, the password to get access to the website and information on how to create an account. To secure standardised instructions, there will be an instruction movie available on the website, (Box 1).

Box 1. The content of the movie, presenting the administration of the COPD Web.

1) Introduction of the website structure, the content in the main headings and functions of the website, e.g., how to enlarge or shrink the text, listen to the text, and bookmark information of particular interest.

2) Introduction to the section "Physical activity." Information about the importance of PA, and demonstration of the page for registration of PA (steps) with automated feedback.

3) Information on how to set an initial weekly step goal and instructions to insert the weekly step-count onto the page for registration of PA at the end of each week.

For peer review only

The COPD Web will be self-managed. To reduce user problems, one of the researchers (TS) will contact each participant in the first week of intervention. To test the participants' interest for and acceptability of the function of registering PA (steps) on the website, the participants will receive a pedometer with instructions on how it is used.

Throughout the intervention, participants will receive prompts via email and SMS (figure 2). The prompts will include targeted information, referral links to the COPD Web, and a reminder to register counted steps to improve adherence to the intervention. Prompts has shown enhanced effectiveness on limited contact interventions targeting health behaviours including PA[30] and proved to be useful also on people with COPD[29] though there is no consensus regarding the number and frequency of prompts. Frequently delivered prompts have been recommended however too excessive appearance may decrease the desired response.[31] Consequently, the frequency of the prompts will be each week at the beginning of the intervention and decrease to biweekly (week 13 to 24) and every fourth week (week 25 to 52). In total, we will deliver 24 different prompts with predetermined content and order to each participant.

Figure 2. Distribution of prompts (SMS and email) to participants in the intervention group.

The control group

The control group will, similar to the intervention group, receive a pedometer with instructions, as well as a leaflet about the importance of PA in addition to usual care. In Sweden, the majority of all people with COPD are treated within PHC.[13 15] Usual care within PHC are recommended to include, but are not restricted to, use of long-acting anticholinergics and long-acting β2-agonists with 24 h duration and support for; smoking cessation, PA and exercise, self-management and nutrition.[13] All participants are permitted to start COPD rehabilitation or other interventions if offered at their PHC unit.

Outcomes and evaluation

Various methods for data collection including questionnaires, accelerometer, data from medical records (participant's latest pulmonary function test), qualitative interviews, and user data from the COPD Web will be used. Table 2 provides an overview of methods for data collection in this study.

Primary outcome measures

The primary outcome of the effect of the COPD Web is the difference in the level of PA between intervention and control groups at follow-ups (3 and 12 months). Level of PA will be objectively measured seven consecutive days using an accelerometer (DynaPort®, McRoberts BV, the

For peer review only

Netherlands) and subjectively measured with indicator questions on PA from the National Board of Health and Welfare in Sweden.[32 33] Weekends and weekdays with less than eight hours of wearing time of the accelerometer and measurements with less than four valid days of measurements will be excluded.[34]The Dynaport accelerometer has been found valid and reliable when used in people with COPD.[34 35]

Secondary outcome measures

The secondary outcomes of the effect of the COPD Web are the differences between the intervention and control groups at the follow-ups at 3 and 12 months regarding participants' dyspnea; modified Medical Research Council dyspnea scale (mMRC)[36], HRQoL; Chronic Respiratory Questionnaire, self-administered (CRQ-SA)[37], and COPD-related symptoms; COPD Assessment Test (CAT).[38] Evaluation of health economics will be done using EQ-5D[39] to estimate quality-adjusted life (QALY) gained, commonly used in economic evaluation.[40] In addition, the number of participant self-reported COPD-related healthcare contacts will be evaluated where a reduction in health consumption indicates a reduced economic burden. Secondary outcomes were chosen according to results in the pilot study and since they cover specific aspects of the content of the COPD Web. Most of them have previously been used in COPD and a Swedish context.

User experience and implementation evaluation

For user experience evaluation, data will be collected after 3 months using semi-structured individual interviews in a subgroup of participants randomised to intervention. The participants will be asked to take part in an interview at 3 months follow-up. The interviews will include questions regarding unexpected events or consequences of receiving the COPD Web, their use of the website, and how this use has influenced their PA behaviour. Study-specific documentation and automatised data on the participants' use of the COPD Web will be collected automatically from the website, e.g., the number of visits, pages used, and time spent on the website. This will add valuable information to the experience valuation but also make it possible to evaluate the fidelity to the intervention. In order to evaluate the implementation and reach, study-specific documentation including the number of participants who decline to take part in the intervention as well as dropouts will be noted. In addition, the reasons to decline will be noted when appropriate. All participants will also answer study-specific questions regarding other ongoing or started interventions, hospitalisations or exacerbations that could affect the results.

For peer review only

Table 2. Methods for data collection.

Physical objectively measured physical activity (PA) level

- Accelerometer (DynaPort, McRoberts BV (DynaPort®, McRoberts BV, The Netherlands) placed on the lower back 24 hours a day over seven consecutive days.[34 35]
  - The quantity of PA will be assessed using the mean number of steps per day and the number of days per week that the participant could be considered physically active. Physically active is operationally defined as ≥5000 steps per day.
  - The Dynaport accelerometer has been found valid and reliable when used in people with COPD.[34 35]

Physical subjectively assessed PA level

- Questionnaire from the National Board of Health and Welfare.[33]
  - The time spent in physical activities such as taking a walk or working in the garden during last week is rated by choosing between pre-specified options (no time at all/30–60 min/60–90 min/9–120 min/>120 min).
  - The time spent in physical exercises such as running or doing exercise to keep fit during last week is rated by choosing between pre-specified options (no time at all/30–60 min/60–90 min/9–120 min/>120 min).
  - The categorical mode of the scale has shown low-to-moderate associations with objectively measured PA level, maximal oxygen uptake, physical performance, balance, cardiovascular biomarkers, and self-rated health.[32]

Health-related quality of life (HRQoL)

- CRQ-SA The Swedish version of the self-administrated Chronic Respiratory Questionnaire.[37]
  - CRQ-SA aims to measure HRQoL in people with chronic respiratory distress. The questionnaire consists of 20 questions divided into four areas (dyspnea, fatigue, emotional function, and control) that are rated on a 7-graded Likert scale. The questions include, for example, "How often in the last two weeks have you known that you had complete control over your breathing problems?" and "In the last two weeks, how often have you known that you had low energy?".[37]
  - CRQ-SA has shown strong responsiveness to changes in HRQoL for people with COPD.[41]

COPD-related symptoms

- The questionnaire COPD Assessment Test (CAT).[38]
  - The severity of eight COPD-related symptoms (coughing, the presence of phlegm, feeling of tightness in the chest, breathlessness when walking, limitation in activities, confidence in leaving home, sleep, and energy) is rated on a six-grade scale.
  - Evaluated for internal consistency, stability over time in stable patients and ability to discriminate between stable and exacerbation patients with excellent or very good results.[38]

Dyspnea

- The questionnaire modified Medical Research Council Dyspnea Scale (mMRC).[36]
  - Perceived dyspnea is rated on a 5-graded Likert scale ranging from 0 ("I just get out of breath when I exert myself greatly" to 4 ("I get out of breath when I wash or get dressed").
  - Evaluated for categorising people with COPD in terms of disability with good results.[42]

Health economics

- Self-reported healthcare contacts related to COPD.
- The questionnaire EuroQol five dimensions questionnaire(EQ-5D).[39]
  - Health status is rated on five items; three items relate to problems in mobility, self-care, and usual activities, and two items cover the presence and severity of pain and anxiety/depression. Each item is rated on a three-grade scale corresponding to no problem/some or moderate problems/extreme problems.

For peer review only

- General health is rated on a scale ranging from 0 (worst imaginable health state) to 100 (best possible health state).
- Evaluation of health economy will be done using EQ-5D to estimate quality-adjusted life (QALY) gained.[40] Also, the number of COPD-related health contacts and hospitalisation that occurs during the intervention will be followed and cost estimated.
- EQ-5D can discriminate between groups of people with different severity of COPD.[43]

Implementation

- Implementation of the COPD Web.
  - Semi-structured interviews will be performed according to a pre-specified interview guide, and user statistics from the website will be analysed.
- Fidelity to the intervention.
  - Semi-structured interviews will be performed according to a pre-specified interview guide.
- Reach.
  - Study-specific documentation including the number of participants who decline to take part in the intervention will be analysed. When appropriate, the reasons to decline will be noted.
- Enablers and barriers for the use of web-based support like the COPD Web.
  - Semi-structured interviews will be performed according to a pre-specified interview guide and analysed.

COPD, chronic obstructive pulmonary disease.

Data collection, management, and analysis

Sample size calculation

The sample size was calculated with the premises that a total of 144 participants with COPD would be required to detect a mean difference of 1131 steps with a standard deviation of 2193 steps[44], $\alpha = 0.05$, $\beta = 0.20$ (80% power), and a two-tailed test of significance including an estimated dropout rate of 20%.[29] Approximately 10-15 participants will be recruited to individual interviews to have various experiences represented. A wide distribution of age, disease severity and an equal number of women and men will be strived for.

Randomisation and masking

A permuted block design with a random block size varying from 4 to 8 in a 1:1 allocation ratio will be computer generated to randomise participants. This approach is chosen to achieve balanced and evenly distributed samples. A third party, not involved in data collection or analysis of the results, will perform the randomisation and the result will be stored in sealed envelopes. Thus, the randomisation will be revealed for the researcher when the baseline registration and written informed consent are fulfilled, and the sealed envelope next in order is opened. The researcher then will send a letter containing the result of group allocation, a pedometer, a pamphlet about PA, and information about when the participant will be contacted again. The members of the intervention group will, in addition, receive the material and information on how to start using the COPD Web.

For peer review only

Due to the character of the intervention, blinding of trial participants will not be applicable. Furthermore, as all data are self-reported, neither is blinding of outcome assessors applicable.

Data management and monitoring

To ensure confidentiality, participants with COPD will get a unique identification (ID) when included in the study. The code list linking participants and ID number will be kept separate from the data. Data will be analysed by ID only. All records that contain names or other personal identifiers, such as locator forms and informed consent forms, will be stored separately from study records identified by the ID number. The local database will be secured with a password-protected access system. All data will be coded and reported on group level. Thus it will not be possible to identify specific participants in the trial. We will use two-pass verification to ensure correct data entry. No interim analyses or stopping guidelines are pre-specified. Only the researchers will have access to the final trial dataset.

Statistics and qualitative analysis

The primary analysis will be an intention-to-treat analysis (including all participants randomised). In addition, a complete case population (participants with full outcome measurements independent on adherence to intervention), and a per-protocol analysis (defined as at least one login besides creating an account on the COPD Web or answering that the SMS and email with referral links have been used at least rarely (1-3 times) at the follow-ups) will be performed. Missing data will be imputed in the intention-to-treat analysis using multiple imputation assuming data is missing at random conditional on participants' severity of disease and self-reported history of exacerbations. This is because the severity of disease and history of exacerbations are known risk factors for future exacerbations and may affect adherence to PA interventions.[45]

The difference in the primary outcome between the intervention and control group will be estimated using multilevel mixed-effects models with subjects at level 1 and PHC units at level 2. PHC units and subjects will be modelled as random effects while group (intervention group vs. control group), time and group*time interaction as fixed effects. Estimates of effect sizes will be computed using Cohen's d (d = difference in group means/error SD within). Calculated as the difference between predicted means from the final mixed-effects model for a given pair of groups divided by the estimated within-group error SD in the model with the estimated value of $2\sigma_e^2$, where σ_e^2 is the residual variance. To judge the quality of the model we, will analyse the residuals. No sub-group or adjusted analyses other than the pre-specified complete case and per-protocol analysis will be performed.

The individual interviews will be analysed using qualitative content analysis according to the procedures presented by Graneheim.[46] The interviews transcriptions will be read, coded, and categorised by one researcher. Two other researchers will also read and code independently for

For peer review only

triangulation. Organisation and labelling of categories will be discussed and modified throughout the process.

Amendments

Any modifications to the protocol that may influence the conduct of the study, the potential benefit of the participant or may affect participant safety, including changes of study objectives, study design, population, sample sizes, study procedures or significant administrative aspects will require a formal amendment to the protocol. Such modifications will be agreed upon by the research group with the final decision by the principal investigator, and if needed to be approved by the local ethics committee.

Administrative changes of the protocol (e.g., minor corrections and clarifications) that do not influence how the study is conducted will be agreed upon by the research group with the final decision by the principal investigator and will be documented and presented upon publication.

Ethics approval and consent to participate

Ethical approval has been received from the Regional Ethical Review Board in Umeå, Sweden. Dnr 2018-274-31. All participants will receive brief, comprehensible oral and written information, by the Helsinki Declaration.[47] A first informed consent confirms that contact information and latest pulmonary function test from the potential participant can be collected by healthcare professionals and sent to the researchers. The participant will, together with the baseline assessment, send a second and final informed consent to the researcher. The informed consent from operational managers will be sent and stored at the Regional Ethical Review Board in Umeå, Sweden.

Dissemination

The results of this study will be submitted for publication in peer-reviewed journals and presented at conferences both nationally and internationally as well as to included healthcare professionals, participants, and patient organisations for people with COPD.

Trial registration

Registration of the clinical trial before the enrolment of the first participant was performed. Date of trial initial release 2018-11-15 and published 2018-12-20. ClinicalTrials.gov identifier: NCT03746873. The recruitment began 2018-11-15 and will continue until sufficient power is reached.

For peer review only

Discussion

This study protocol presents a pragmatic randomised controlled trial with pre- and post-assessments aimed at evaluating the effect of the use of the COPD Web for people with COPD in a PHC context. The study also intends to evaluate the implementation and to identify enablers and barriers to use of web-based support to change behaviour among people with COPD. Currently, despite its proven effectiveness, access to self-management interventions is limited[2 14], and alternative ways of promoting self-management for people with COPD are warranted. A recent pilot trial has shown that giving people with COPD access to the COPD Web may be an effective short-term strategy to promote self-management that increase levels of PA, promote conceptual knowledge and alter disease management strategies.[24] However, these results need to be confirmed in a definitive large-scale randomised trial, including both short- and long-term evaluation.

This proposed trial will provide new knowledge to this research area by evaluating the effect of the use of web-based support for increasing access to self-management strategies for people with COPD and determine its effect on clinically relevant outcomes. This trial will include short- (3 months) and long-term perspectives (12 months) with objectively measured PA in addition to the self-reported PA that will contribute with more knowledge regarding the effect of having access to the COPD Web. PA is of utmost importance, as the level of PA is one of the strongest predictors of mortality among people with COPD.[11 12]

A user experience and implementation evaluation of the intervention will provide novel information and understanding about enablers and barriers for the use of web-based support to change behaviour. This information will increase knowledge of how the process of receiving the intervention can be interpreted. It will also help us draw better conclusions regarding acceptance, fidelity, and implementation of the COPD Web.

Guided by the pilot study, prompts will be used to encourage the use of the website during the intervention period.[24] The reminders will provide information with referral links that will appear in a predefined way. Prompts have been proven effective in other setups, but there is no consensus regarding the number of prompts or frequency, especially in a longer perspective.[31] The effect of the prompts will be qualitatively evaluated through the semi-structured interviews. The evaluation will answer how the prompts were perceived and if they induced more frequent use and/or changed behaviour regarding PA among the participants. The use of the COPD Web will be automatically registered through the whole intervention since the participants need to log in to access the website. That measure makes it possible to analyse the fidelity to the intervention and answer if there is an

For peer review only

association between the use of the COPD Web, e.g., time and number of visits and any possible effect.

As the study is designed as a pragmatic trial[25], the intervention will be self-managed and distance-based to maximise the clinical applicability of the findings. One concern is that there might be participants who do not manage the instructions to create their account and learn how to use the website. However, they will be contacted at the beginning of the intervention to reduce user problems. The pragmatic approach also means that there is no selection on the number, size, or location of the recruiting PHC units. Also, the inclusion criteria are set wide with a minimised selection beyond diagnosed COPD that could enhance the recruitment rates and finally increase the clinical applicability of the findings within PHC. One limitation is that the sample size, calculated on PA, will be large enough for evaluation of the PA but may not be powered enough for all secondary outcome or sub-group analyses. The latter much depending on the severity of symptoms among the participants.

In conclusion, this pragmatic randomised trial will provide clinically relevant information on the effect of the use of the COPD Web in people with COPD in a PHC context regarding level of PA, dyspnea, HRQoL, COPD-related symptoms and health economics in relation to healthcare use, as well as barriers and enablers for using web-based support with solutions such as the COPD Web.

Ethics approval and consent to participate

Regional Ethical Review Board in Umeå, Sweden.

Availability of data and materials

Not applicable.

Consent for publication

Not applicable.

Competing interests

AN reports lecture fees from AstraZeneca.

Author Contributions

TS has made a direct and substantial contribution to this work by contributing to the conception and design of the study, designing and writing of the protocol. AN has made a direct and substantial

For peer review only

contribution to this work by contributing to the conception and design of the study, sample size calculation and choice of statistics, designing and writing of the protocol. SL has made a direct and substantial contribution to this work in providing critical revisions that are important for the intellectual content of the protocol. KW is the principal investigator and has made a direct and substantial contribution to this work by providing the project idea, contributing to the conception and design of the study and by providing critical revisions that are important for the intellectual content of the protocol. All authors have approved the final version of the protocol.

Acknowledgement

Not applicable

Funding

This work was supported by The Swedish Research Council, grant number 521-2013-3503 and the Strategic Research Area – Care Science, Umeå University, Sweden, no grant number available.

References

1. World Health Organization. The top 10 causes of death.: http://www.who.int/mediacentre/factsheets/fs310/en/; [accessed 2019-03-19].
2. Jansson SA, Backman H, Stenling A, et al. Health economic costs of COPD in Sweden by disease severity - Has it changed during a ten years period? *Respir Med* 2013 doi: 10.1016/j.rmed.2013.07.012
3. Jansson SA, Backman H, Ronmark E, et al. Hospitalization Due to Co-Morbid Conditions is the Main Cost Driver Among Subjects With COPD-A Report From the Population-Based OLIN COPD Study. *Copd* 2015;12(4):381-9. doi: 10.3109/15412555.2014.974089 [published Online First: 2014/11/22]
4. Vogelmeier CF, Criner GJ, Martinez FJ, et al. Global Strategy for the Diagnosis, Management, and Prevention of Chronic Obstructive Lung Disease 2017 Report. GOLD Executive Summary. *Am J Respir Crit Care Med* 2017;195(5):557-82. doi: 10.1164/rccm.201701-0218PP [published Online First: 2017/01/28]
5. Spruit MA, Singh SJ, Garvey C, et al. An official American Thoracic Society/European Respiratory Society statement: key concepts and advances in pulmonary rehabilitation. *Am J Respir Crit Care Med* 2013;188(8):e13-64. doi: 10.1164/rccm.201309-1634ST [published Online First: 2013/10/17]
6. Effing TW, Bourbeau J, Vercoulen J, et al. Self-management programmes for COPD: moving forward. *Chronic respiratory disease* 2012;9(1):27-35. doi: 10.1177/1479972311433574
7. Apps LD, Mitchell KE, Harrison SL, et al. The development and pilot testing of the self-management programme of activity, coping and education for chronic obstructive pulmonary disease (SPACE for COPD). *International journal of chronic obstructive pulmonary disease* 2013;8:317-27. doi: 10.2147/COPD.S40414
8. Velloso M, Jardim JR. Study of energy expenditure during activities of daily living using and not using body position recommended by energy conservation techniques in patients with COPD. *Chest* 2006;130(1):126-32. doi: 10.1378/chest.130.1.126

For peer review only

9. Garcia-Aymerich J, Pitta F. Promoting Regular Physical Activity in Pulmonary Rehabilitation. *Clinics in Chest Medicine* 2014;35(2):363-68. doi: 10.1016/j.ccm.2014.02.011
10. Troosters T, Sciurba F, Battaglia S, et al. Physical inactivity in patients with COPD, a controlled multi-center pilot-study. *Respir Med* 2010;104(7):1005-11. doi: 10.1016/j.rmed.2010.01.012 [published Online First: 2010/02/20]
11. Waschki B, Kirsten A, Holz O, et al. Physical activity is the strongest predictor of all-cause mortality in patients with COPD: a prospective cohort study. *Chest* 2011;140(2):331-42. doi: 10.1378/chest.10-2521 [published Online First: 2011/01/29]
12. Gimeno-Santos E, Frei A, Steurer-Stey C, et al. Determinants and outcomes of physical activity in patients with COPD: a systematic review. *Thorax* 2014;69(8):731-9. doi: 10.1136/thoraxjnl-2013-204763
13. Socialstyrelsen [The National Board of Health and Welfare]. Nationella riktlinjer för vård vid astma och KOL 2018. [National guidelines for asthma and COPD care] [accessed March 13, 2019].
14. Wadell K, Janaudis Ferreira T, Arne M, et al. Hospital-based pulmonary rehabilitation in patients with COPD in Sweden-A national survey. *Respir Med* 2013;107(8):1195-200. doi: 10.1016/j.rmed.2013.04.019
15. Sundh J, Lindgren H, Hasselgren M, et al. Pulmonary rehabilitation in COPD - available resources and utilization in Swedish primary and secondary care. *International journal of chronic obstructive pulmonary disease* 2017;12:1695-704. doi: 10.2147/copd.s135111 [published Online First: 2017/06/28]
16. Lundell S, Holmner A, Rehn B, et al. Telehealthcare in COPD: A systematic review and meta-analysis on physical outcomes and dyspnea. *Respir Med* 2015;109(1):11-26. doi: 10.1016/j.rmed.2014.10.008
17. Loeckx M, Rabinovich RA, Demeyer H, et al. Smartphone-Based Physical Activity Telecoaching in Chronic Obstructive Pulmonary Disease: Mixed-Methods Study on Patient Experiences and Lessons for Implementation. *JMIR mHealth and uHealth* 2018;6(12):e200. doi: 10.2196/mhealth.9774
18. Adams SG, Smith PK, Allan PF, et al. Systematic review of the chronic care model in chronic obstructive pulmonary disease prevention and management. *Archives of internal medicine* 2007;167(6):551-61. doi: 10.1001/archinte.167.6.551
19. Moy ML, Collins RJ, Martinez CH, et al. An Internet-Mediated Pedometer-Based Program Improves Health-Related Quality-of-Life Domains and Daily Step Counts in COPD: A Randomized Controlled Trial. *Chest* 2015;148(1):128-37. doi: 10.1378/chest.14-1466 [published Online First: 2015/03/27]
20. Wan ES, Kantorowski A, Homsy D, et al. Promoting physical activity in COPD: Insights from a randomized trial of a web-based intervention and pedometer use. *Respir Med* 2017;130:102-10. doi: 10.1016/j.rmed.2017.07.057 [published Online First: 2017/12/06]
21. McCabe C, McCann M, Brady AM. Computer and mobile technology interventions for self-management in chronic obstructive pulmonary disease. *Cochrane Database of Systematic Reviews* 2017(5) doi: 10.1002/14651858.CD011425.pub2
22. Tistad M, Lundell S, Wiklund M, et al. Usefulness and Relevance of an eHealth Tool in Supporting the Self-Management of Chronic Obstructive Pulmonary Disease: Explorative Qualitative Study of a Cocreative Process. *JMIR Hum Factors* 2018;5(4):e10801. doi: 10.2196/10801 [published Online First: 2018/10/29]
23. Nyberg A, Wadell K, Lindgren H, et al. Internet-based support for self-management strategies for people with COPD-protocol for a controlled pragmatic pilot trial of effectiveness and a process evaluation in primary healthcare. *BMJ open* 2017;7(7):e016851. doi: 10.1136/bmjopen-2017-016851 [published Online First: 2017/08/03]
24. Nyberg A, Tistad M, Wadell K. Can the COPD web be used to promote self-management in patients with COPD in swedish primary care: a controlled pragmatic pilot trial with 3 month-

For peer review only

and 12 month follow-up. *Scandinavian journal of primary health care* 2019:1-14. doi: 10.1080/02813432.2019.1569415 [published Online First: 2019/02/01]

25. Zwarenstein M, Treweek S, Gagnier JJ, et al. Improving the reporting of pragmatic trials: an extension of the CONSORT statement. *BMJ (Clinical research ed)* 2008;337:a2390. doi: 10.1136/bmj.a2390 [published Online First: 2008/11/13]
26. Chan A-W, Tetzlaff JM, Altman DG, et al. SPIRIT 2013 Statement: Defining Standard Protocol Items for Clinical Trials. *Annals of Internal Medicine* 2013;158(3):200-07. doi: 10.7326/0003-4819-158-3-201302050-00583
27. Chan AW, Tetzlaff JM, Gotzsche PC, et al. SPIRIT 2013 explanation and elaboration: guidance for protocols of clinical trials. *BMJ (Clinical research ed)* 2013;346:e7586. doi: 10.1136/bmj.e7586 [published Online First: 2013/01/11]
28. Eysenbach G. CONSORT-EHEALTH: Improving and Standardizing Evaluation Reports of Web-based and Mobile Health Interventions. *J Med Internet Res* 2011;13(4):e126. doi: 10.2196/jmir.1923 [published Online First: 31.12.2011]
29. Demeyer H, Louvaris Z, Frei A, et al. Physical activity is increased by a 12-week semiautomated telecoaching programme in patients with COPD: a multicentre randomised controlled trial. *Thorax* 2017;72(5):415-23. doi: 10.1136/thoraxjnl-2016-209026
30. Fry JP, Neff RA. Periodic Prompts and Reminders in Health Promotion and Health Behavior Interventions: Systematic Review. *J Med Internet Res* 2009;11(2):e16. doi: 10.2196/jmir.1138 [published Online First: 14.5.2009]
31. Muench F, Baumel A. More Than a Text Message: Dismantling Digital Triggers to Curate Behavior Change in Patient-Centered Health Interventions. *J Med Internet Res* 2017;19(5):e147. doi: 10.2196/jmir.7463 [published Online First: 2017/05/28]
32. Olsson SJG, Ekblom Ö, Andersson E, et al. Categorical answer modes provide superior validity to open answers when asking for level of physical activity: A cross-sectional study. *Scandinavian Journal of Public Health* 2016;44(1):70-76. doi: 10.1177/1403494815602830
33. Socialstyrelsen [The National Board of Health and Welfare]. Nationella riktlinjer för sjukdomsförebyggande metoder 2011 Tobaksbruk, riskbruk av alkohol, otillräcklig fysisk aktivitet och ohälsosamma matvanor Stöd för styrning och ledning [Disease Prevention in the Swedish Healthcare System: Health situation, national guidelines and implementation]. (In Swedish with an English summary). Västerås, Sweden: Socialstyrelsen, 2011. .
34. Demeyer H, Burtin C, Van Remoortel H, et al. Standardizing the analysis of physical activity in patients with COPD following a pulmonary rehabilitation program. *Chest* 2014;146(2):318-27. doi: 10.1378/chest.13-1968 [published Online First: 2014/03/08]
35. Andersson M, Janson C, Emtner M. Accuracy of three activity monitors in patients with chronic obstructive pulmonary disease: a comparison with video recordings. *Copd* 2014;11(5):560-7. doi: 10.3109/15412555.2014.898033 [published Online First: 2014/04/17]
36. Mahler DA, Wells CK. Evaluation of clinical methods for rating dyspnea. *Chest* 1988;93(3):580-6. [published Online First: 1988/03/01]
37. Vernooij RW, Willson M, Gagliardi AR. Characterizing patient-oriented tools that could be packaged with guidelines to promote self-management and guideline adoption: a meta-review. *Implementation science : IS* 2016;11:52. doi: 10.1186/s13012-016-0419-1 [published Online First: 2016/04/16]
38. Jones PW, Harding G, Berry P, et al. Development and first validation of the COPD Assessment Test. *The European respiratory journal* 2009;34(3):648-54. doi: 10.1183/09031936.00102509 [published Online First: 2009/09/02]
39. Dolan P. Modeling valuations for EuroQol health states. *Medical care* 1997;35(11):1095-108. [published Online First: 1997/11/21]
40. Manca A, Hawkins N, Sculpher MJ. Estimating mean QALYs in trial-based cost-effectiveness analysis: the importance of controlling for baseline utility. *Health Economics* 2005;14(5):487-96. doi: 10.1002/hec.944

For peer review only

41. Puhan MA, Guyatt GH, Goldstein R, et al. Relative responsiveness of the Chronic Respiratory Questionnaire, St. Georges Respiratory Questionnaire and four other health-related quality of life instruments for patients with chronic lung disease. *Respiratory Medicine* 2007;101(2):308-16. doi: https://doi.org/10.1016/j.rmed.2006.04.023
42. Bestall JC, Paul EA, Garrod R, et al. Usefulness of the Medical Research Council (MRC) dyspnoea scale as a measure of disability in patients with chronic obstructive pulmonary disease. *Thorax* 1999;54(7):581-6. [published Online First: 1999/06/22]
43. Rutten-van Molken MP, Oostenbrink JB, Tashkin DP, et al. Does quality of life of COPD patients as measured by the generic EuroQol five-dimension questionnaire differentiate between COPD severity stages? *Chest* 2006;130(4):1117-28. doi: 10.1378/chest.130.4.1117 [published Online First: 2006/10/13]
44. Demeyer H, Burtin C, Hornikx M, et al. The Minimal Important Difference in Physical Activity in Patients with COPD. *PLOS ONE* 2016;11(4):e0154587. doi: 10.1371/journal.pone.0154587
45. Müllerová H, Shukla A, Hawkins A, et al. Risk factors for acute exacerbations of COPD in a primary care population: a retrospective observational cohort study. *BMJ open* 2014;4(12):e006171. doi: 10.1136/bmjopen-2014-006171
46. Graneheim UH, Lundman B. Qualitative content analysis in nursing research: concepts, procedures and measures to achieve trustworthiness. *Nurse education today* 2004;24(2):105-12. doi: 10.1016/j.nedt.2003.10.001 [published Online First: 2004/02/11]
47. World Medical A. World medical association declaration of helsinki: Ethical principles for medical research involving human subjects. *JAMA* 2013;310(20):2191-94. doi: 10.1001/jama.2013.281053

For peer review only

Table 1. Participant timeline for enrolment, the intervention and assessments.

Timepoint	t^{-1} screening/consent	t^{0} baseline	t^{1} start	t^{2} 3 months	t^{3} (interviews)	t^{4} 12 months
**Enrolment**						
Eligibility screen	x					
Informed consent		x				
Allocation			x			
**Intervention**						
The COPD Web			→	→	→	→
**Assessments**						
Sociodemographic (age, sex, anthropometry, diagnosis)[1]		x		x		x
Pulmonary function[2]	x					
COPD-related symptoms[1]		x		x		x
Dyspnea[1]		x		x		x
Health-related quality of life (HRQoL)[1]		x		x		x
Time spent in physical activity and training[1]		x		x		x
Time being sedentary[1]		x		x		x
Physical activity level (accelerometer)[1]		x		x		x
Implementation [1,3]			x	x	x	x
Response to and interaction with the COPD Web[1]				x	x	x
COPD-related health care contacts[1]				x		x
Enablers and Barriers for the use of a web-based solution[1]					x	

Data collection from [1] People with COPD, [2] Medical records, [3] Statistics from the website.

For peer review only

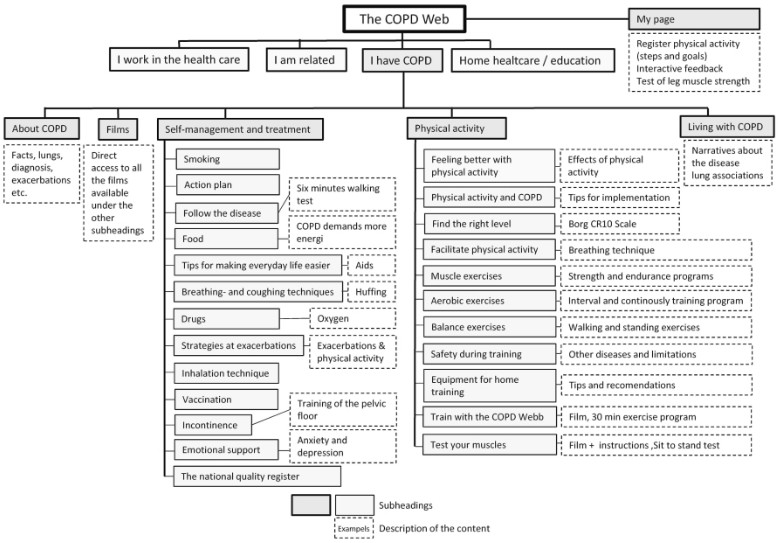

Figure 1. A website map of the COPD Web showing the section “I have COPD”.

297x209mm (300 x 300 DPI)

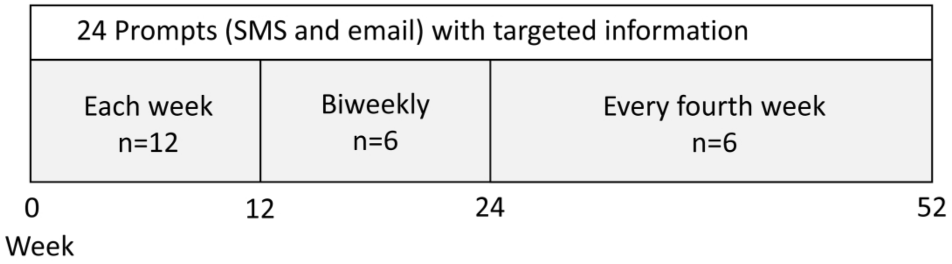

Figure 2. Distribution of prompts (SMS and email) to participants in the intervention group

183x57mm (600 x 600 DPI)

Reporting checklist for protocol of a clinical trial.

Based on the SPIRIT guidelines.

Instructions to authors

Complete this checklist by entering the page numbers from your manuscript where readers will find each of the items listed below.

Your article may not currently address all the items on the checklist. Please modify your text to include the missing information. If you are certain that an item does not apply, please write "n/a" and provide a short explanation.

Upload your completed checklist as an extra file when you submit to a journal.

In your methods section, say that you used the SPIRIT reporting guidelines, and cite them as:

Chan A-W, Tetzlaff JM, Altman DG, Laupacis A, Gøtzsche PC, Krleža-Jerić K, Hróbjartsson A, Mann H, Dickersin K, Berlin J, Doré C, Parulekar W, Summerskill W, Groves T, Schulz K, Sox H, Rockhold FW, Rennie D, Moher D. SPIRIT 2013 Statement: Defining standard protocol items for clinical trials. Ann Intern Med. 2013;158(3):200-207

		Reporting Item	Page Number
Title	#1	Descriptive title identifying the study design, population, interventions, and, if applicable, trial acronym	1
Trial registration	#2a	Trial identifier and registry name. If not yet registered, name of intended registry	2+13
Trial registration: data set	#2b	All items from the World Health Organization Trial Registration Data Set	n/a
Protocol version	#3	Date and version identifier	n/a
Funding	#4	Sources and types of financial, material, and other support	15
Roles and responsibilities: contributorship	#5a	Names, affiliations, and roles of protocol contributors	1+15-16

For peer review only

Roles and responsibilities: sponsor contact information	#5b	Name and contact information for the trial sponsor	n/a
Roles and responsibilities: sponsor and funder	#5c	Role of study sponsor and funders, if any, in study design; collection, management, analysis, and interpretation of data; writing of the report; and the decision to submit the report for publication, including whether they will have ultimate authority over any of these activities	15
Roles and responsibilities: committees	#5d	Composition, roles, and responsibilities of the coordinating centre, steering committee, endpoint adjudication committee, data management team, and other individuals or groups overseeing the trial, if applicable (see Item 21a for data monitoring committee)	n/a
Background and rationale	#6a	Description of research question and justification for undertaking the trial, including summary of relevant studies (published and unpublished) examining benefits and harms for each intervention	4
Background and rationale: choice of comparators	#6b	Explanation for choice of comparators	4
Objectives	#7	Specific objectives or hypotheses	5
Trial design	#8	Description of trial design including type of trial (eg, parallel group, crossover, factorial, single group), allocation ratio, and framework (eg, superiority, equivalence, non-inferiority, exploratory)	5+11
Study setting	#9	Description of study settings (eg, community clinic, academic hospital) and list of countries where data will be collected. Reference to where list of study sites can be obtained	6
Eligibility criteria	#10	Inclusion and exclusion criteria for participants. If applicable, eligibility criteria for study centres and individuals who will perform the interventions (eg, surgeons, psychotherapists)	5-6

For peer review only

Interventions: description	#11a	Interventions for each group with sufficient detail to allow replication, including how and when they will be administered	7-8
Interventions: modifications	#11b	Criteria for discontinuing or modifying allocated interventions for a given trial participant (eg, drug dose change in response to harms, participant request, or improving / worsening disease)	13
Interventions: adherance	#11c	Strategies to improve adherence to intervention protocols, and any procedures for monitoring adherence (eg, drug tablet return; laboratory tests)	8
Interventions: concomitant care	#11d	Relevant concomitant care and interventions that are permitted or prohibited during the trial	n/a
Outcomes	#12	Primary, secondary, and other outcomes, including the specific measurement variable (eg, systolic blood pressure), analysis metric (eg, change from baseline, final value, time to event), method of aggregation (eg, median, proportion), and time point for each outcome. Explanation of the clinical relevance of chosen efficacy and harm outcomes is strongly recommended	8-10
Participant timeline	#13	Time schedule of enrolment, interventions (including any run-ins and washouts), assessments, and visits for participants. A schematic diagram is highly recommended (see Figure)	6-7+20
Sample size	#14	Estimated number of participants needed to achieve study objectives and how it was determined, including clinical and statistical assumptions supporting any sample size calculations	11
Recruitment	#15	Strategies for achieving adequate participant enrolment to reach target sample size	6
Allocation: sequence generation	#16a	Method of generating the allocation sequence (eg, computer-generated random numbers), and list of any factors for stratification. To reduce predictability of a random sequence, details of any planned restriction (eg, blocking) should be provided in a separate document that	11

For peer review only

		is unavailable to those who enrol participants or assign interventions	
Allocation concealment mechanism	#16b	Mechanism of implementing the allocation sequence (eg, central telephone; sequentially numbered, opaque, sealed envelopes), describing any steps to conceal the sequence until interventions are assigned	11
Allocation: implementation	#16c	Who will generate the allocation sequence, who will enrol participants, and who will assign participants to interventions	11
Blinding (masking)	#17a	Who will be blinded after assignment to interventions (eg, trial participants, care providers, outcome assessors, data analysts), and how	12
Blinding (masking): emergency unblinding	#17b	If blinded, circumstances under which unblinding is permissible, and procedure for revealing a participant's allocated intervention during the trial	n/a
Data collection plan	#18a	Plans for assessment and collection of outcome, baseline, and other trial data, including any related processes to promote data quality (eg, duplicate measurements, training of assessors) and a description of study instruments (eg, questionnaires, laboratory tests) along with their reliability and validity, if known. Reference to where data collection forms can be found, if not in the protocol	8-11
Data collection plan: retention	#18b	Plans to promote participant retention and complete follow-up, including list of any outcome data to be collected for participants who discontinue or deviate from intervention protocols	7
Data management	#19	Plans for data entry, coding, security, and storage, including any related processes to promote data quality (eg, double data entry; range checks for data values). Reference to where details of data management procedures can be found, if not in the protocol	12
Statistics: outcomes	#20a	Statistical methods for analysing primary and secondary outcomes. Reference to where other details of the statistical analysis plan can be found, if not in the protocol	12-13

For peer review only

Statistics: additional analyses	#20b	Methods for any additional analyses (eg, subgroup and adjusted analyses)	12
Statistics: analysis population and missing data	#20c	Definition of analysis population relating to protocol non-adherence (eg, as randomised analysis), and any statistical methods to handle missing data (eg, multiple imputation)	12
Data monitoring: formal committee	#21a	Composition of data monitoring committee (DMC); summary of its role and reporting structure; statement of whether it is independent from the sponsor and competing interests; and reference to where further details about its charter can be found, if not in the protocol. Alternatively, an explanation of why a DMC is not needed	n/a
Data monitoring: interim analysis	#21b	Description of any interim analyses and stopping guidelines, including who will have access to these interim results and make the final decision to terminate the trial	12
Harms	#22	Plans for collecting, assessing, reporting, and managing solicited and spontaneously reported adverse events and other unintended effects of trial interventions or trial conduct	9
Auditing	#23	Frequency and procedures for auditing trial conduct, if any, and whether the process will be independent from investigators and the sponsor	n/a
Research ethics approval	#24	Plans for seeking research ethics committee / institutional review board (REC / IRB) approval	2+13+15
Protocol amendments	#25	Plans for communicating important protocol modifications (eg, changes to eligibility criteria, outcomes, analyses) to relevant parties (eg, investigators, REC / IRBs, trial participants, trial registries, journals, regulators)	13
Consent or assent	#26a	Who will obtain informed consent or assent from potential trial participants or authorised surrogates, and how (see Item 32)	6+12-13

For peer review only

Consent or assent: ancillary studies	#26b	Additional consent provisions for collection and use of participant data and biological specimens in ancillary studies, if applicable	n/a
Confidentiality	#27	How personal information about potential and enrolled participants will be collected, shared, and maintained in order to protect confidentiality before, during, and after the trial	12
Declaration of interests	#28	Financial and other competing interests for principal investigators for the overall trial and each study site	14
Data access	#29	Statement of who will have access to the final trial dataset, and disclosure of contractual agreements that limit such access for investigators	12
Ancillary and post trial care	#30	Provisions, if any, for ancillary and post-trial care, and for compensation to those who suffer harm from trial participation	n/a
Dissemination policy: trial results	#31a	Plans for investigators and sponsor to communicate trial results to participants, healthcare professionals, the public, and other relevant groups (eg, via publication, reporting in results databases, or other data sharing arrangements), including any publication restrictions	13
Dissemination policy: authorship	#31b	Authorship eligibility guidelines and any intended use of professional writers	n/a
Dissemination policy: reproducible research	#31c	Plans, if any, for granting public access to the full protocol, participant-level dataset, and statistical code	n/a
Informed consent materials	#32	Model consent form and other related documentation given to participants and authorised surrogates	n/a
Biological specimens	#33	Plans for collection, laboratory evaluation, and storage of biological specimens for genetic or molecular analysis in the current trial and for future use in ancillary studies, if applicable	n/a

The SPIRIT checklist is distributed under the terms of the Creative Commons Attribution License CC-BY-ND 3.0. This checklist was completed on 29. March 2019 using https://www.goodreports.org/, a tool made by the [EQUATOR Network]() in collaboration with [Penelope.ai]()

For peer review only
